# The Novel MuRF2 Target SNX5 Regulates PKA Activity Through Stabilization of RI‐α and Controls Myogenic Differentiation

**DOI:** 10.1002/jcsm.70103

**Published:** 2025-10-12

**Authors:** Ning Li, Jida Hamati, Yi Li, Björn Brinschwitz, Mohamed Ghait, Elisa Martin, Dörte Lodka, Elke Hammer, Britta Fielitz, Uwe Völker, Gunnar Dittmar, Thomas Sommer, Friedrich C. Luft, Jens Fielitz

**Affiliations:** ^1^ DZHK (German Center for Cardiovascular Research), partner site Greifswald Greifswald Germany; ^2^ Department of Internal Medicine B, Cardiology University Medicine Greifswald Greifswald Germany; ^3^ Experimental and Clinical Research Center, Charité Universitätsmedizin Berlin Max Delbrück Center for Molecular Medicine in the Helmholtz Association Berlin Germany; ^4^ Interfaculty Institute for Genetics and Functional Genomics University Medicine Greifswald Greifswald Germany; ^5^ Luxembourg Institute of Health Strassen Luxembourg; ^6^ Department of Life Sciences and Medicine University of Luxembourg Belvaux Luxembourg; ^7^ Max Delbrück Center for Molecular Medicine in the Helmholtz Association Berlin Germany; ^8^ Institute for Biology Humboldt‐University zu Berlin Berlin Germany; ^9^ DZHK (German Center for Cardiovascular Research), partner site Berlin Berlin Germany

**Keywords:** muscle RING‐finger protein, myostatin, protein kinase A, RI‐α, sorting nexin 5

## Abstract

**Background:**

Muscle RING finger (MuRF) proteins are striated muscle‐specific E3 ubiquitin ligases essential for muscle homeostasis. Whereas MuRF1 is well known for its role in muscle atrophy, MuRF2 and MuRF3 contribute to microtubule stabilization, influencing muscle differentiation and function. Their cooperative functions in regulating myogenesis are unclear. This study aimed to identify novel MuRF2 and MuRF3 interaction partners and investigate their function in myogenic differentiation.

**Methods:**

Interaction partners of MuRF2 and MuRF3 were identified using stable isotope labelling with amino acids in cell culture (SILAC), followed by affinity purification and quantitative mass spectrometry (AP‐MS). Mechanistic analyses included co‐immunoprecipitation, domain mapping, ubiquitination assays, protein stability measurements and endosome isolation. Myogenic differentiation was evaluated by immunocytochemistry, qRT‐PCR and western blotting. Functional effects were assessed using CRISPR‐Cas9‐mediated knockout and siRNA silencing.

**Results:**

We identified sorting nexin 5 (SNX5), a BAR and PX domain‐containing retromer component involved in retrograde vesicular transport, as a novel MuRF2 and MuRF3 binding partner. Both coiled‐coil domains of MuRF3 were required for SNX5 binding, and the BAR domain of SNX5 mediated interaction with MuRF2 and MuRF3. Immunofluorescence staining demonstrated MuRF3–SNX5 interaction and colocalization on early endosomes along microtubules in myocytes. MuRF2 promoted ubiquitination of SNX5 at lysines 290 and 324, leading to proteasomal degradation, whereas MuRF3 counteracted this effect. Mass spectrometry revealed the protein kinase A regulatory subunit (PKA‐RI‐α) as cargo of SNX5‐coated early endosomes in myocytes. SNX5 knockout (SNX5‐KO) reduced RI‐α stability in myocytes, enhanced PKA activity and increased HDAC5 degradation via the autophagy‐lysosomal pathway, leading to MEF2‐mediated upregulation of myostatin. SNX5‐KO impaired myogenesis, with significant reductions in myogenin/*Myog* (*p* < 0.005), myomaker/*Mymk* (*p* < 0.01), myomerger/*Mymx* (*p* < 0.005) and MyHC isoforms *Myh2* and *Myh4* (*p* < 0.01). Myostatin treatment mimicked the SNX5‐KO phenotype, reducing fast‐twitch MyHC isoforms *Myh1*, *Myh2*, *Myh3* and *Myh4* (*p* < 0.05 for all) and significantly lowering Myomaker, Myomerger and MyHC expression throughout differentiation (*p* < 0.05 for all). Morphologically, myostatin‐treated cells were shorter and thinner and had fewer nuclei. Quantification showed reduced differentiation and fusion indices (*p* < 0.001) and fewer nuclei per myosin‐positive cell (*p* < 0.01).

**Conclusions:**

MuRF2 and MuRF3 exert opposing effects on SNX5‐mediated retrograde transport, influencing PKA signalling and myogenic differentiation. SNX5 stabilizes RI‐α within early endosomes, facilitating ordered myogenic differentiation. Our findings expand the known functions of MuRF proteins beyond proteasomal degradation and identify SNX5 as a key regulator of PKA activity in muscle cells. These insights may provide novel therapeutic targets for muscle‐related disorders.

## 1 Introduction

Muscle‐specific ‘really interesting new gene’ (RING)‐finger (MuRF) proteins are a family of E3 ubiquitin ligases uniquely expressed in striated muscle, where they regulate protein homeostasis, metabolism and transcription. MuRF1 is the most studied and a key player in skeletal muscle atrophy [1]. The functions of MuRF2 and MuRF3 are less defined. MuRF2 regulates cardiomyocyte signal transduction via serum response transcription factor (SRF) degradation [S1], whereas MuRF3 binds to contractile and structural proteins, such as myosin heavy chain (MyHC), mediates their ubiquitination and degradation via the 26S proteasome [2]. MuRF3 also maintains cardiac integrity post‐myocardial infarction, as *Trim54* (MuRF3) knockout mice develop heart failure and cardiac rupture following ischaemic injury [2]. MuRF proteins function cooperatively by binding to shared substrates [3, 4]. This is evident in combined germ‐line *Trim63*/*Trim55* (MuRF1/MuRF2) [S2, S3], *Trim55*/*Trim54* (MuRF2/MuRF3) [5] and *Trim63*/*Trim54* (MuRF1/MuRF3) [2] knockout mice, which results in cardiac and skeletal muscle myopathies. In contrast, single knockouts of *Trim63* [S4], *Trim55* [6] and *Trim54* [7] show no major phenotype. Notably, *Trim63*/*Trim55* double knockout (DKO) mice died perinatally [S2, S3], whereas *Trim55*/*Trim54* and *Trim63*/*Trim54* DKO mice show accumulations of MyHC proteins, leading to protein‐surplus myopathies [2, 5]. However, the functional differences between MuRF2 and MuRF3 regarding their shared substrates remain unknown.

Beyond their role in the ubiquitin–proteasome system (UPS), MuRF2 and MuRF3 bind to and stabilize microtubules, crucial for maintaining cellular structure and myogenic differentiation [8, 9]. Both proteins link microtubules to sarcomeric proteins, a function essential for cargo transport via endosome trafficking and retrograde transport [10]. Whether MuRFs influence vesicle transport independently of microtubule stabilization remains uncertain. Cargo internalized via endocytosis is either recycled, degraded or transported retrogradely to the trans‐Golgi network (TGN), a process mediated by the retromer complex [11, 12]. A key retromer component, sorting nexin 5 (SNX5), binds phosphatidylinositol 3‐phosphate (PtdIns(3)P) on endosomal membranes via its phox‐homology (PX) domain and interacts with other SNX family members [13, 14].

Protein kinase A is a serine/threonine kinase critical for muscle homeostasis and myogenic differentiation [15]. In its inactive state, PKA exists as a heterotetramer composed of a regulatory dimer (RI‐α) bound to a catalytic dimer [16]. Upon cyclic AMP (cAMP) stimulation, RI‐α dissociates, activating PKA activity [17]. PKA activity is tightly regulated, partly via RI‐α degradation, which enhances PKA activation [17, 18]. The vesicular uptake of RI‐α can regulate PKA activity [19]. Notably, RI‐α that is not internalized is subject to degradation, leading to sustained PKA activation [20]. Whether SNX5‐coated EE influences RI‐α stability and PKA activity in myocytes, and whether this impacts myogenic differentiation, remains unknown.

Here, we identified SNX5 as a novel MuRF2 and MuRF3 interaction partner. MuRF2, but not MuRF3, promotes SNX5 ubiquitination and degradation. We show that RI‐α is a cargo within SNX5‐coated EE. In SNX5‐deficient cells, RI‐α degradation increases, leading to heightened PKA activity, enhanced CREB phosphorylation and altered gene expression, including decreased acetylcholinesterase (*Ache*) and increased myostatin (*Mstn*). Additionally, elevated PKA activity reduces HDAC5, thereby enhancing MEF2‐dependent myostatin upregulation and impairing myogenic differentiation.

Our findings reveal opposing roles for MuRF2 and MuRF3 in SNX5 regulation. We propose that MuRF proteins contribute to SNX5‐mediated retrograde transport of RI‐α, thereby modulating PKA activity and myogenic differentiation via CREB and the HDAC5/MEF2 axis.

## 2 Materials and Methods

### 2.1 Animals

Tissues from mice of our previously published work [21] were used to quantitate SNX5 mRNA and protein expression levels. All animal procedures were performed in accordance with the guidelines of the Max‐Delbrück Center for Molecular Medicine and were approved by the Regional Office for Health and Social Affairs Berlin (G0129/12). They followed the principles of laboratory animal care set forth by the National Institutes of Health (NIH) in the Guide for the Care and Use of Laboratory Animals (NIH Publication 86‐23, revised 1985).

### 2.2 Stable Isotope Labeling by Amino Acids in Cell Culture (SILAC)

#### 2.2.1 SILAC Labeling of C2C12 Cells

To identify novel interaction partners of MuRF3, we used stable isotope labelling by amino acids in cell culture (SILAC), combined with affinity purification and mass spectrometry (SILAC‐AP‐MS; Figure S1). This approach enables direct comparison of two experimental conditions within a single mass spectrometry run, thereby increasing quantitative accuracy and minimizing technical variability compared to label‐free shogun proteomics [S5, S6]. SILAC improves sensitivity for detecting MuRF3‐specific interaction partners, especially low‐abundance or transient interactors, and allows efficient discrimination between true interactors and non‐specific background binders.

We established two isotope‐labelled C2C12 cell lines. One line was cultured in medium containing unlabelled lysine (Lys0), whereas a second was grown in medium supplemented with a non‐radioactive, isotopically labelled form of lysine, called Lys4. Cells were maintained in SILAC growth medium (SILAC DMEM, 4.5 g/L glucose [without glutamine, lysine and arginine]) (Qiagen, Germany) supplemented with 10% dialyzed FBS (Sigma‐Aldrich, Germany; cut‐off for dialysis membrane: 10 kDa), 2 mM L‐glutamine, 20 μg/mL L‐arginine, 100 U/mL penicillin and 100 μg/mL streptomycin. For SILAC labelling, Lys0 or Lys4 (Linde AG, Germany) was added to final concentrations of 4 and 50 μg/mL, respectively. Dialyzed FBS was chosen as it is devoid of free amino acids that would otherwise interfere with isotope incorporation. To ensure efficient isotope incorporation, C2C12 cells were cultivated in SILAC media for at least five passages. Mass spectrometric analysis confirmed labelling efficiencies exceeding 97%, indicating successful replacement of endogenous lysine by Lys4 (data not shown). Labelled cells were maintained in SILAC media until further usage.

For the pulldown experiment, Lys0‐labelled C2C12 cells were transiently transfected with MuRF3‐Myc (His)_6_, whereas Lys4‐labelled cells received control vector (pcDNA3.1(‐)A‐Myc (His)_6_). After 24 h, cells were lysed in lysis buffer (10 mM Tris–HCl (pH 7.5), 150 mM NaCl and protease inhibitors). Lysis was performed by three freeze–thaw cycles followed by mechanical shearing using a syringe fitted with a 0.40 × 20 mm needle. Lysates were first cleared by centrifugation at 300 g for 10 min, and the supernatants further clarified by ultracentrifugation at 50 000 rpm for 20 min. The resulting soluble fraction was subjected to affinity purification. For pulldown, 50 μL of Ni‐Sepharose‐6 Fast Flow (Sigma‐Aldrich, Germany) was washed three times with ice‐cold PBS and incubated with cell lysates in a total volume of 1.5 mL for 3 h at 4°C under gentle rotation at 15 rpm. Proteins were eluted by resuspension of the sepharose in 100 μL of 120 mM EDTA (pH 7.4) at room temperature. Eluates from Lys0‐ and Lys4‐labelled cells were combined and stored at −80°C until mass spectrometric analysis. Proteins enriched in Lys0‐MuRF3 samples relative to Lys4‐control were considered putative MuRF3 interaction partners.

### 2.3 CRISPR/Cas9‐Mediated Gene Editing

CRISPR/Cas9 gene editing was used to delete *Snx5* in C2C12 cells according to the manufacturer's instructions (IDT, Integrated DNA Technologies Inc., USA). Briefly, the trancrRNA (Cat‐No. 1073190) was mixed with either scrambled negative control crRNA (Cat‐No. 1079138, both IDT, USA) or SNX5‐targeting crRNA (sequences are shown in Table S1). The mixture was heated at 95°C for 5 min. The ribonucleoprotein (RNP) complex was formed by adding the Cas9‐eGFP protein (Cat‐No. 10008100, IDT, USA) followed by a 5‐min incubation at room temperature. RNAiMAX (Thermo Fisher Scientific, USA) was used to transfect the RNP complex into C2C12 myoblasts. Twenty‐four hours after transfection, the BD FACSAria III Cell Sorter (BD Biosciences, USA) was used to sort for eGFP‐positive single cells, which were propagated in GM to obtain *Snx5* deleted single cell clones. Deletion of *Snx5* was confirmed by sequencing, and absence of SNX5 protein was confirmed by immunoblotting.

Other materials and methods can be found in the Supporting Information.

## 3 Statistics

All experiments were performed independently and at least three times using biological triplicates each until stated otherwise. Statistical analysis was performed using GraphPad Prism 7 (GraphPad Software Inc., USA). Differences between two groups were evaluated with an unpaired two‐tailed Student's *t*‐test. One‐way analysis of variance (ANOVA) followed by Tukey's post hoc test was used for the comparison of more than two independent groups with only one factor. Data are presented as mean ± standard deviation. Photoshop and Illustrator (both Adobe, USA) and FIJI/ImageJ software (Wayne Rasband, National Institutes of Health, USA) were used for plots. *p* < 0.05 was considered statistically significant.

## 4 Results

### 4.1 SNX5 Is a Novel MuRF2 and MuRF3 Interaction Partner

To identify novel MuRF3 interaction partners, we performed a SILAC‐based affinity purification coupled with mass spectrometry (SILAC‐AP‐MS) in C2C12 myoblasts transfected with His‐tagged MuRF3 or control plasmids (workflow in Figure S1A). Several MuRF3‐associated proteins were identified (Table S2), including those linked to microtubule dynamics and vesicular transport [8, 9, 22]. Notably, the retromer subunit sorting nexin 5 (SNX5) was significantly enriched in MuRF3 precipitates (Table S2). SNX5 is a key component of retrograde vesicular transport of endosomes to the TGN, mainly associated with EE [14, S7], but its role in myocytes was previously unexplored.

**FIGURE 1:**
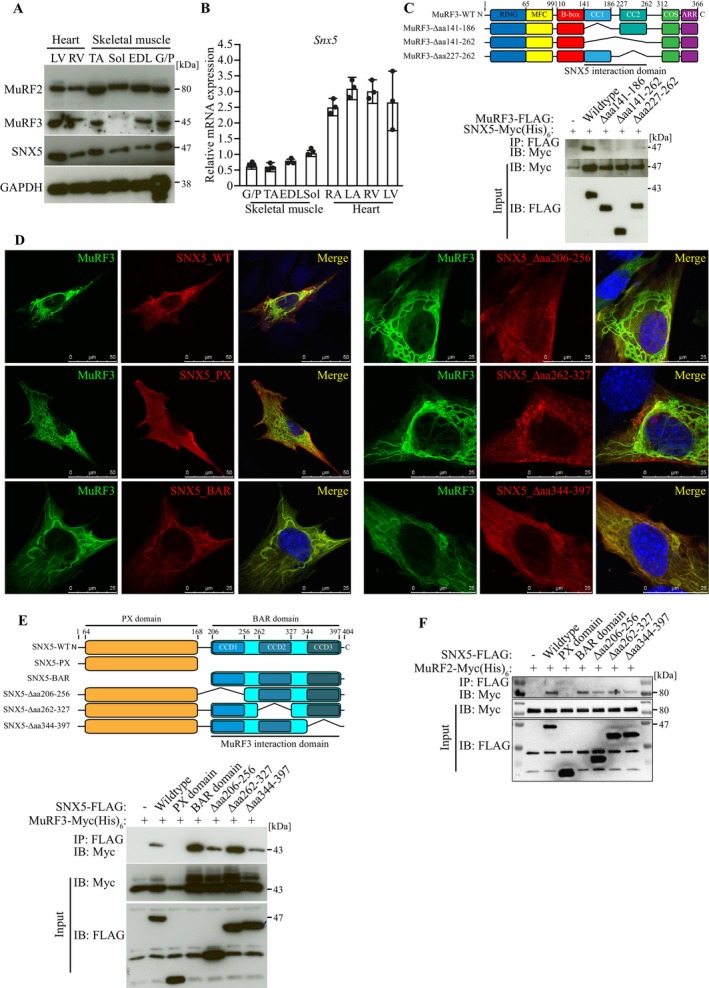
The retromer subunit sorting nexin 5 is a novel MuRF2 and MuRF3 interaction partner. (A) Western blot and (B) qRT‐PCR analysis of the indicated proteins and *Snx5* mRNAs isolated from murine heart (right atrium [RA], left atrium [LA], right ventricle [RV], left ventricle [LV]) and skeletal muscle (gastrocnemius and plantaris [G/P], tibialis anterior [TA], extensor digitorum longus [EDL], soleus [Sol]). *Snx5* mRNA expression was normalized to *Gapdh*. (C) Schematic of MuRF3 domain organization with deletion mutants used for Co‐IP to determine the MuRF3 interaction domain. MFC indicates MuRF‐family conserved domain; ARR, acidic rich region; CC1, coiled coil domain 1; CC2, coiled coil domain 2; COS, C‐terminal subgroup one signature. Co‐immunoprecipitation (Co‐IP) using lysates from HEK293 cells transfected with the indicated expression plasmids. Lysates were immunoprecipitated with anti‐FLAG M2 agarose, and western blotting was performed to detect Myc and FLAG. (D) Immunofluorescence analysis using anti‐Myc (green) and anti‐FLAG (red) antibodies to detect MuRF3‐Myc (His)_6_, SNX5‐FLAG, SNX5‐BAR‐FLAG, SNX5‐PX‐FLAG, SNX5‐Δaa206‐256‐FLAG, SNX5‐Δaa262‐327‐FLAG and SNX5‐Δaa344‐397‐FLAG in transfected C2C12 cells. Nuclei were stained with DAPI. Scale bar, 25 μm. (E) Schematic of SNX5 domain organization with deletion mutants used for immunofluorescence and Co‐IP to determine the SNX5 interaction domain. Co‐IP using lysates from HEK293 cells co‐transfected with indicated plasmids. Lysates were immunoprecipitated with anti‐FLAG M2 agarose, and western blotting was performed to detect Myc and FLAG. (F) Co‐IP using lysates from COS‐7 cells transfected with the indicated expression plasmids. Lysates were immunoprecipitated with anti‐FLAG M2 agarose, and western blotting was performed to detect Myc and FLAG.

Because MuRF proteins are uniquely expressed in striated muscles [9, 22], we analysed SNX5 mRNA and protein expression in muscle tissues. qRT‐PCR and western blot analyses confirmed SNX5 expression in heart and skeletal muscle (Figure 1A,B) as well as non‐muscle tissues (Figure S1B,C). Co‐immunoprecipitation and immunofluorescence staining demonstrated MuRF3–SNX5 interaction and colocalization in myocytes (Figure 1C,D). Domain‐specific MuRF3 mutants (Figure 1C) revealed that both coiled‐coil (CC) domains were required for SNX5 binding.

SNX5 contains a Phox homology (PX) domain and a BAR domain, the latter composed of three CC domains (CC1, CC2 and CC3) (Figure 1E). Co‐immunoprecipitation using SNX5 mutants revealed that only the BAR domain mediated MuRF3 interaction (Figure 1E). Immunofluorescence confirmed that MuRF3 colocalized with SNX5‐BAR along microtubule‐like structures in C2C12 myocytes (Figure 1D). Because MuRF2 and MuRF3 cooperate functionally [5], we tested whether MuRF2 also interacts with SNX5. Co‐immunoprecipitation confirmed MuRF2–SNX5 interaction via the BAR domain (Figure 1F). Collectively, these findings identify SNX5 as a novel MuRF2 and MuRF3 interaction partner.

### 4.2 MuRF2 Targets SNX5 for Ubiquitin Proteasome–Dependent Degradation

**FIGURE 2:**
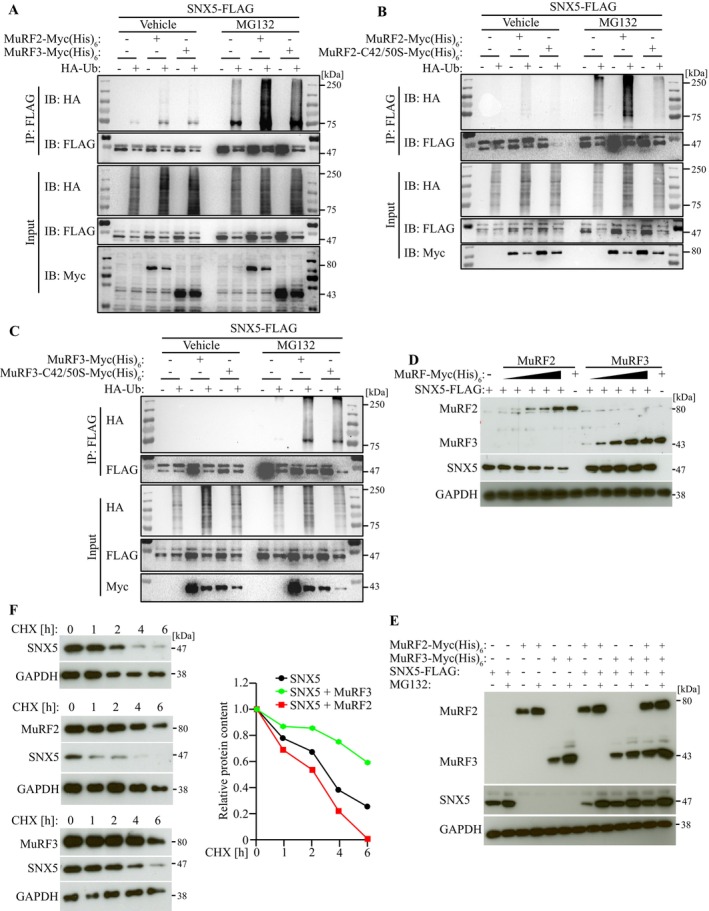
MuRF2 but not MuRF3 ubiquitinates and reduces the stability of SNX5. (A–C) Ubiquitination assays: COS‐7 cells were transfected with indicated plasmids for 42 h, followed by treatment with MG132 (25 μM) or vehicle (0.25% DMSO) for 6 h. Immunoprecipitation with anti‐FLAG M2 agarose was followed by western blotting for HA, Myc and FLAG. (D,E) Western blot analysis of C2C12 cells co‐transfected with SNX5‐FLAG and increasing amounts of MuRF2‐ or MuRF3‐Myc (His)_6_ or MuRF2‐[(C42S; C50S)]‐Myc (His)_6_. (F) CHX chase assay in C2C12 cells co‐transfected with SNX5‐FLAG, MuRF2‐Myc (His)_6_ or MuRF3‐Myc (His)_6_, followed by CHX (100 μg/mL) treatment for indicated time points. Protein contents were analysed by western blot analysis. Densitometrical analysis (right panel) was carried out in Image Lab software. The ‘0 h’ intensity of SNX5 was set as 1. SNX5 protein content was normalized to GAPDH.

As MuRF2 and MuRF3 function as E3 ubiquitin ligases [5], we examined their role in SNX5 ubiquitination. Ubiquitination assays showed that MuRF2 (Figure 2A), but not MuRF3, increased SNX5 ubiquitination. A RING‐finger inactive MuRF2 mutant failed to ubiquitinate SNX5 (Figure 2B), confirming RING‐finger dependency. Neither MuRF3 nor its RING‐finger mutant affected SNX5 ubiquitination (Figure 2C), indicating SNX5 is not a MuRF3 substrate. MuRF2 overexpression decreased SNX5 protein levels in C2C12 myocytes (Figure 2D), an effect absent with MuRF3 (Figure 2D) and MuRF2's RING‐finger mutant (Figure S2A). Coexpression of MuRF3 attenuated MuRF2‐mediated SNX5 reduction in a dose‐dependent manner (Figure 2E and Figure SB). Cycloheximide chase assays confirmed that MuRF2 decreased SNX5 half‐life, whereas MuRF3 stabilized it (Figure 2F).

**FIGURE 3:**
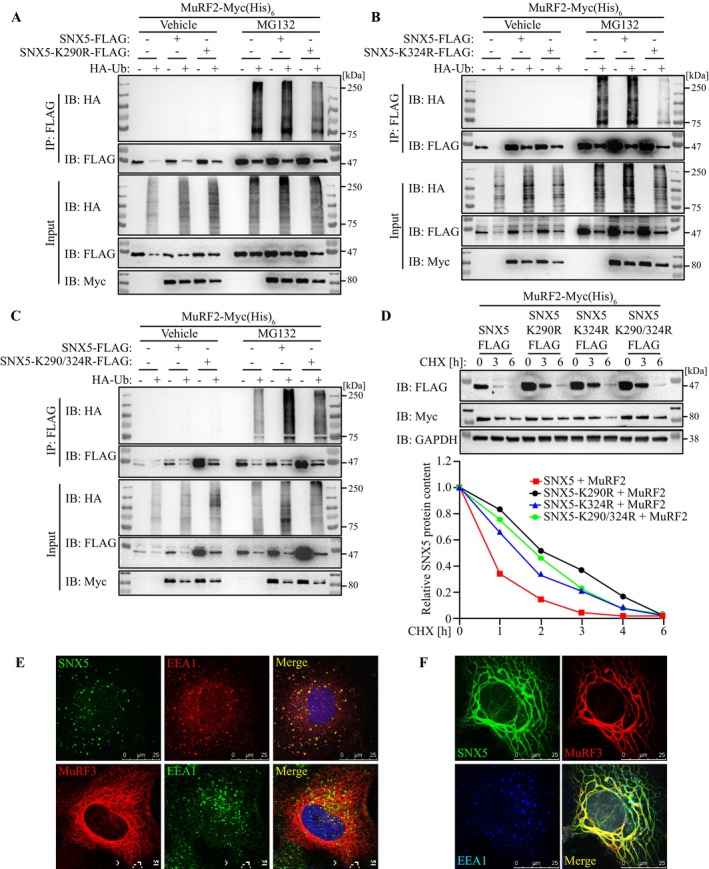
MuRF2‐dependent SNX5 ubiquitination at K290 and K324 mediates its degradation. (A–C) Ubiquitination assays: COS‐7 cells were transfected with FLAG‐SNX5 wild‐type, K290R, K324R, K290/324R mutants, MuRF2‐Myc (His)_6_ and HA‐Ub for 42 h. Cells were treated with MG132 (25 μM) or vehicle for 6 h, followed by immunoprecipitation with anti‐FLAG M2 agarose and western blotting for HA, Myc and FLAG. (D) CHX chase assay in COS‐7 cells co‐transfected with MuRF2‐Myc (His)_6_ and FLAG‐SNX5 wild‐type or K290R, K324R or K290/324R mutants, followed by CHX treatment for indicated time points. Protein contents were analysed by western blot analysis. Densitometrical analysis (lower panel) was carried out in Image Lab software. The ‘0 h’ intensity of SNX5 was set as 1. SNX5 protein content was normalized to GAPDH. (E) Immunofluorescence using anti‐FLAG and anti‐EEA1 antibody to detect FLAG‐SNX5 (green) and early endosome antigen (EEA1) (red) in transfected COS‐7 cells (upper panel). Immunofluorescence using anti‐EEA1 to detect EEA1 (green) and MuRF3‐Cherry (red) in transfected COS‐7 cells (lower panel). Nuclei were stained with DAPI. Scale bar, 25 μm. (F) Immunofluorescence using anti‐FLAG and anti‐EEA1 antibody to detect FLAG‐SNX5 (green), MuRF3‐Cherry (red) and EEA1 (blue) in transfected COS‐7 cells. Scale bar, 25 μm.

Using the AraUbiSite online tool [S8], we identified lysine residues K290 and K324 within SNX5's BAR domain as potential ubiquitination sites (Figure S3A,B). Mutation of these residues (K290R, K324R and K290/324R) significantly reduced MuRF2‐mediated ubiquitination and degradation (Figure 3A–D, Figure S3C). These data establish SNX5 as a MuRF2 substrate in myocytes, with MuRF3 counteracting MuRF2‐dependent SNX5 degradation.

### 4.3 PKA‐RI‐α Is a Novel Cargo of SNX5‐Coated Early Endosomes

To assess SNX5's role in cargo‐selective transport, we examined colocalization with the EE marker EEA1 in C2C12 cells. Immunocytochemistry confirmed SNX5 presence in EE (Figure 3E). As shown previously, MuRF3 colocalized to microtubular‐like structures (Figure 3E) [5, 9] and EEA1 also associated with MuRF3 (Figure 3E). Immunocytochemistry further confirmed the colocalization of SNX5 and MuRF3 and showed that EEA1‐containing EE is located in close vicinity to microtubular‐like structures (Figure 3F).

**FIGURE 4:**
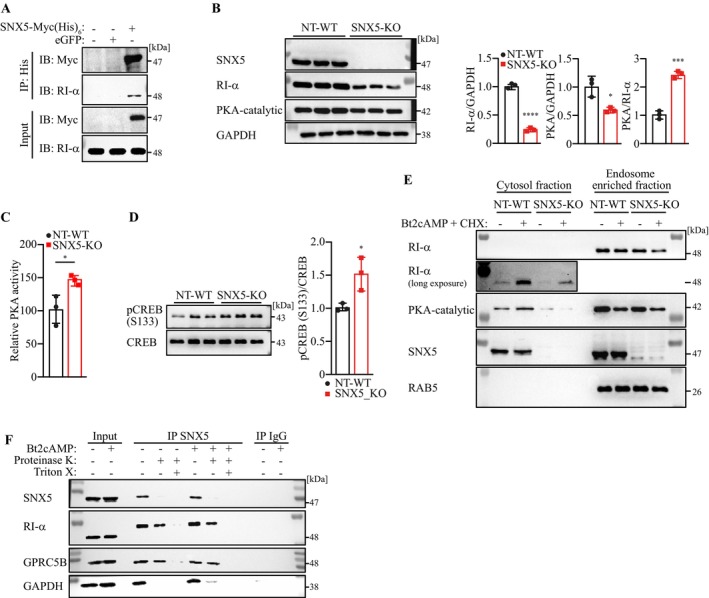
SNX5 mediates retrograde vesicular transport that is accompanied by stabilization of RI‐α. (A) Co‐IP of SNX5‐Myc (His)_6_ or eGFP control vector transduced C2C12 cells, with Nickel‐NTA IP and western blot detection of Myc and RI‐α. (B) Western blot of total lysates from NT‐WT and SNX5‐KO C2C12 cells with indicated antibodies. Densitometrical analysis (lower panel) was carried out in Image Lab software. SNX5, RI‐α and PKA protein contents were normalized to GAPDH. (C) PKA activity assay in NT‐WT and SNX5‐KO C2C12 cells (PKA activity in NT‐WT was set to 100%). (D) Western blot analysis of p‐CREB (Ser133) and CREB in NT‐WT and SNX5‐KO cells. Densitometrical analysis (right panel) was carried out in Image Lab software. p‐CREB‐to‐CREB ratios are shown. (E) Western blot of cytosol and endosome‐enriched fractions from vehicle‐ or Bt2cAMP and CHX co‐treated cells. (F) SNX5‐coated endosomes isolated from vehicle‐ or Bt2cAMP treated C2C12 cells by IP SNX5 were digested with proteinase K in the presence or absence of Triton X‐100 for 1 h at 4°C. Proteins were the immunoblotted as indicated. For (B‐D) data were analysed with unpaired two‐tailed Student's *t*‐test. *p < 0.05, ***p < 0.001, ****p < 0.0001.

Mass spectrometry of SNX5‐coated vesicles that were immunoprecipitated from C2C12 myocytes (workflow in Figure S4A) identified the known SNX5 interactors SNX1 and SNX2 [S9] (Table S3), validating the approach. Notably, RI‐α (cAMP‐dependent protein kinase type I alpha regulatory subunit), a key protein kinase A (PKA) regulator, was enriched (Table S3). Co‐immunoprecipitation confirmed SNX5–RI‐α interaction (Figure 4A). Given the critical role of PKA in muscle growth and differentiation, we focused subsequent analyses on RI‐α and PKA. PKA is composed of two catalytic and two regulatory subunits, the latter serving as cAMP receptors [20]. Upon cAMP binding, regulatory subunits dissociate from the tetramer, activating PKA and triggering downstream phosphorylation events [23]. Interestingly, among the four known PKA regulatory subunits, only RI‐α, but not RI‐β, RII‐α or RII‐β, was enriched in SNX5 precipitates. Sucrose density gradient ultracentrifugation of PNS followed by western blot analysis confirmed that SNX5 and RI‐α co‐fractionate with EEA1‐positive EE in C2C12 cells (Figure S4B).

### 4.4 Upon PKA Activation RI‐α Dissociates From the PKA Catalytic Subunit and Is Stabilized by SNX5

PKA activation triggers RI‐α dissociation from PKA catalytic subunits, leading to phosphorylation of the transcription factor cAMP response element‐binding protein (CREB) at Ser133 [24]. We hypothesized that SNX5 interacts with free cytoplasmic RI‐α and facilitates its uptake into EE, thereby protecting RI‐α from degradation. CRISPR/Cas9‐mediated SNX5 knockout (SNX5‐KO) in C2C12 myocytes (Figure 4B) reduced RI‐α protein but increased the PKA/RI‐α ratio, suggesting elevated PKA activity when compared to non‐targeting controls (NT‐WT). Indeed, SNX5‐KO cells exhibited higher PKA activity and increased pSer133 CREB levels (Figure 4C,D). To verify that PKA activation results in dissociation of RI‐α from the PKA tetramer, we treated C2C12 cells with the PKA activator Bt2cAMP (dibutyryl cyclic adenosine monophosphate) and conducted chemical crosslinking of cell lysates with DSS. We found that PKA activation caused a decrease in the PKA tetramer to the lysate RI‐α ratio. These data indicate that RI‐α dissociated from the PKA tetramer upon PKA activation (Figure S4C).

Immunofluorescence analysis showed RI‐α colocalization with EEA1‐positive EE in NT‐WT but not SNX5‐KO cells (Figure S4D). Endosomal fractionation (workflow in Figure S4E) revealed reduced RI‐α levels in RAB5‐positive EE from SNX5‐KO cells following PKA activation (Figure 4E), supporting SNX5's role in RI‐α stabilization.

A protease protection assay (workflow in Figure S4F) demonstrated that RI‐α is internalized in SNX5‐coated EE, similar to the known endosomal cargo GPRC5B (G protein–coupled receptor class C group 5 member B, [S10]) (Figure 4F). A molecular weight shift in vesicular RI‐α suggested post‐translational modification. Peptide:N‐glycosidase F treatment confirmed RI‐α N‐glycosylation (Figure S4G), implicating this modification in EE localization.

**FIGURE 5:**
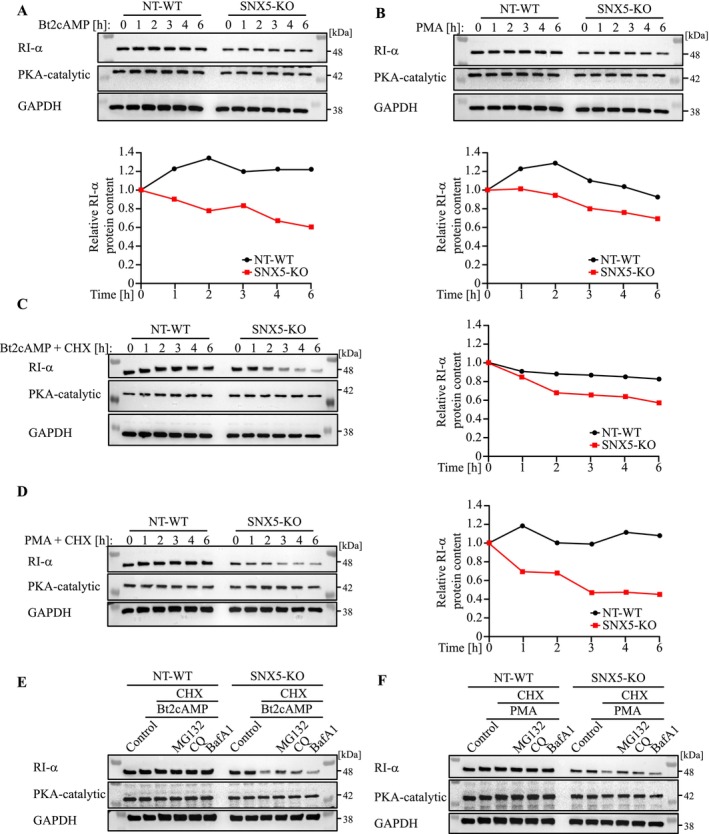
SNX5 stabilizes RI‐α upon PKA activation. (A,B) Western blot of NT‐WT and SNX5‐KO C2C12 cells treated with Bt2cAMP (A) or PMA (B) for the indicated time points. Densitometrical analysis (lower panel) was carried out in Image Lab software. Protein amounts of RI‐α were normalized to GAPDH and RI‐α‐to‐GAPDH ratios per indicated time point are shown. (C,D) Western blot of NT‐WT and SNX5‐KO cells co‐treated with Bt2cAMP (C) or PMA (D) and CHX for the indicated time points. Densitometrical analysis (right panel) was carried out in Image Lab software. Protein amounts of RI‐α were normalized to GAPDH and RI‐α‐to‐GAPDH ratios per indicated time point are shown. (E,F) Western blot of NT‐WT and SNX5‐KO cells co‐treated with Bt2cAMP (E), PMA (F) and CHX and either MG132, CQ or BafA1 for 4 h.

PKA activation with Bt2cAMP and PMA (phorbol‐12‐myristate‐13‐acetate) reduced RI‐α stability in SNX5‐KO but not NT‐WT cells (Figure 5A,B). Increased *Prkar1a* gene expression in SNX5‐KO cells indicated a compensatory mechanism (Figure S5). Cycloheximide chase assays confirmed reduced RI‐α stability post‐PKA activation in SNX5‐KO cells, compared to NT‐WT cells (Figure 5C,D). Reintroduction of SNX5 into SNX5‐KO cells restored RI‐α stability following PKA activation (Figure S6). Proteasome (MG132) and lysosome (CQ) inhibitors attenuated RI‐α degradation in SNX5‐KO cells, whereas autophagy inhibition (BafA1) had no effect (Figure 5E,F). Similar findings were observed upon SNX5 knockdown in C2C12 myoblasts (Figure S7A) and myotubes (Figure S7B), as well as a in heterozygous SNX5‐knockout myocytes (Figure S8). These data suggest SNX5 stabilizes RI‐α by preventing its proteasomal and lysosomal degradation.

### 4.5 SNX5‐Mediated Regulation of PKA Activity Is Involved in Myogenic Differentiation

**FIGURE 6:**
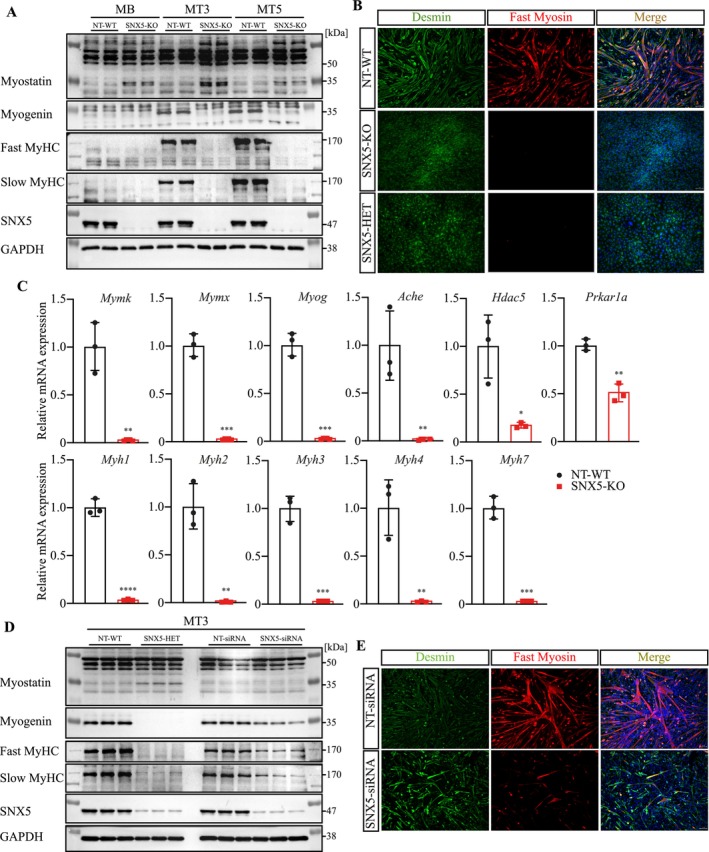
SNX5 is involved in myogenic differentiation. (A) Western blot of proteins isolated from NT‐WT and SNX5‐KO C2C12 myoblasts and three (MT3) and five (MT5) days differentiated myotubes with indicated antibodies. (B) Immunofluorescence detecting Desmin (green) and Fast‐myosin heavy chain (red) in NT‐WT, SNX5‐KO and SNX5‐HET myotubes day 3. Nuclei were stained with DAPI. Scale bar, 100 μm. (C) qRT‐PCR analysis of *Mymk*, *Mymx*, *Myog*, *Ache*, *Hdac5, Prkar1a*, *Myh1*, *Myh2*, *Myh3*, *Myh4* and *Myh7* expression in 3 days differentiated NT‐WT and SNX5‐KO myocytes. mRNA expression of indicated genes was normalized to *Gapdh*. (D) Western blot of lysates from 3 days differentiated NT‐WT and SNX5‐HET myocytes, as well as NT‐siRNA and SNX5‐siRNA transfected C2C12 cells with indicated antibodies. (E) Immunofluorescence of 3‐day differentiated NT‐siRNA and SNX5‐siRNA transfected myotubes detecting Desmin (green) and Fast‐myosin heavy chain (red). Nuclei were stained with DAPI. Scale bar, 100 μm. Data in (C) were analysed using unpaired two‐tailed Student's *t*‐test. *p < 0.05, **p < 0.01, ***p < 0.001, ****p < 0.0001.

PKA activation inhibits skeletal myogenesis via myostatin induction [25]. Myostatin is produced by skeletal muscle and acts on myofibres and muscle stem cells (i.e., satellite cells) [26]. It reduces muscle mass by increasing protein degradation and inhibiting protein synthesis [26] and inhibits myogenic differentiation [27]. Western blot analysis revealed that myostatin levels were increased in SNX5‐KO myocytes (Figure 6A). Immunocytochemistry showed a defective myogenic differentiation of SNX5‐KO myocytes (Figure 6B). Western blot analysis revealed a decrease in the myogenic transcription factor Myogenin and a reduction of the terminal differentiation markers slow and fast MyHC in differentiating SNX5‐KO myocytes, compared to NT‐WT myocytes (Figure 6A). The expression of myogenic markers *Myogenin/Myog*, *Myomaker/Mymk*, *Myomerger/Mymx* several *MyHC/Myh*, as well as *Ache*, *Hdac5* and *Prkar1a* was greatly reduced in differentiating SNX5‐KO myocytes (Figure 6C). We confirmed these findings in heterozygous SNX5‐knockout myocytes (Figure 6B,D), as well as in C2C12 myotubes, where SNX5 was reduced by siRNA (Figure 6D,E).

**FIGURE 7:**
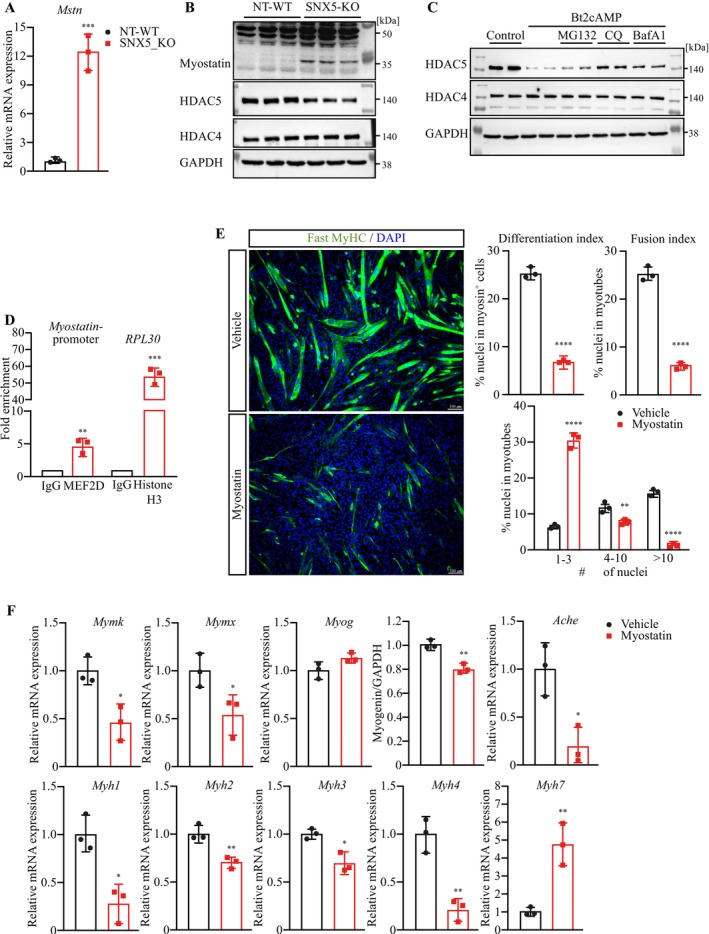
SNX5 via regulation of PKA activity contributes to myogenic differentiation. (A) qRT‐PCR analysis of *Mstn* mRNA expression from NT‐WT and SNX5‐KO myoblasts. *Mstn* mRNA expression was normalized to *Gapdh*. (B) Western blot analysis of cell lysates from NT‐WT and SNX5‐KO C2C12 cells with indicated antibodies. (C) Western blot analysis of C2C12 cells treated with Bt2cAMP for 66 h and further co‐treatment with either MG132, CQ or BafA1 as indicated for 6 h prior to cell lysis. (D) Chromatin immunoprecipitation (ChIP) enrichment assay. ChIP fold enrichment of DNA fragments around myostatin promotor region was analysed by qRT‐PCR. (E) Immunofluorescence detecting Fast‐myosin heavy chain (green) in differentiated myotubes treated with recombinant myostatin or vehicle for 3 days. Nuclei were stained with DAPI. Scale bar, 100 μm. Differentiation index, fusion index and nuclei distribution in each myosin^+^–myocyte were quantified from images (right panel). (F) qRT‐PCR analysis of *Mymk*, *Mymx*, *Myog*, *Ache*, *Myh1*, *Myh2*, *Myh3*, *Myh4* and *Myh7* mRNA expression from vehicle or recombinant myostatin‐treated C2C12 myotubes (MT3). mRNA expression of indicated genes was normalized to *Gapdh*. Data (A, E [upper panel] and F) were analysed with unpaired two‐tailed Student's *t*‐test. Data (D and E [lower panel]) were analysed with one‐way ANOVA followed by Tukey's post hoc test. *p < 0.05, **p < 0.01, ***p < 0.001, ****p < 0.0001.

We next investigated the mechanism how SNX5 regulates myogenic differentiation. Activated PKA causes an increased CREB phosphorylation and nuclear translocation, where it promotes Myostatin (*Mstn*) and represses *Ache* expression [28], thereby inhibiting myogenic differentiation [25]. In SNX5‐KO myoblasts, we observed an increased Myostatin mRNA and protein expression (Figures 6A and 7A,B). Furthermore, PKA has also been shown to affect Class IIa histone deacetylases (HDACs), particularly HDAC4 and HDAC5. These HDACs repress the transcription factor myocyte enhancer factor 2 (MEF2) and reduce myogenesis [29]. Western blot analyses showed a reduction in HDAC5, but not HDAC4 protein levels in SNX5‐KO myocytes compared to NT‐WT myocytes (Figure 7B). To investigate if PKA activation contributes to the reduction in HDAC5, we activated PKA by Bt2cAMP and quantitated *Hdac5* expression in myocytes at different time points. PKA activation from 12 to 72 h led to a reduction in *Hdac5* expression, which was not observed with short‐term PKA activation (Figure S9A), indicating that the reduction in HDAC5 occurs at both mRNA and protein levels. We next tested if SNX5 affects PKA‐induced nuclear accumulation of HDAC5 and found less HDAC5 in the nuclei of SNX5‐KO compared to NT‐WT myocytes (Figure S9B). CHX chase assays confirmed that nuclear HDAC5 is less stable in the absence of SNX5 in myocytes (Figure S9B). Our data also suggest that nuclear HDAC5 is destabilized in the absence of SNX5 (Figure S9B). The reduction in HDAC5 in response to PKA activation was reversed by the lysosomal inhibitors, CQ and BafA1. This result indicates that PKA mediates HDAC5 degradation via the autophagy‐lysosome pathway, whereas HDAC4 remained unaffected (Figure 7C). HDAC5 was shown to inhibit MEF2D activity [29], which was reported to increase *Mstn* expression [S11]. We therefore hypothesized that the reduction of HDAC5 enhances MEF2‐mediated *Mstn* expression. CHIP‐qPCR analysis revealed that MEF2D was indeed bound to the *Mstn* promoter in myocytes, indicating that MEF2D promotes *Mstn* expression and that a reduction in HDAC5 can increase this effect (Figure 7D).

To further assess myostatin's role in myogenic differentiation, we treated C2C12 myoblasts with recombinant myostatin during differentiation. Western blot analysis, qRT‐PCR and immunocytochemistry revealed that myostatin‐treated myotubes expressed more slow‐twitch (MyHC and *Myh7*) and less fast‐twitch MyHC (*Myh1*, *Myh2*, *Myh3* and *Myh4*) (Figure 7E,F and Figure S9C), were thinner and shorter and comprised fewer nuclei when compared with vehicle‐treated cells on differentiation day 3 (Figure 7E). Quantification of differentiation and fusion indices and the number of nuclei per myosin‐positive cells showed that myostatin attenuated myogenic differentiation (Figure 7E). qRT‐PCR demonstrated that myostatin‐treated cells showed a reduction of the differentiation markers Myomaker/*Mymk*, Myomerger/*Mymx* and fast‐twitch MyHC/*Myh* throughout differentiation (Figure 7F). In summary, these data demonstrate that myostatin attenuates both myoblast fusion and myogenic differentiation.

## 5 Discussion

The MuRF proteins are E3‐ubiquitin ligases that regulate muscle growth and turnover, yet their individual roles remain incompletely defined. Our findings suggest that MuRF proteins interact to regulate SNX5 in muscle homeostasis. Specifically, SNX5‐mediated stabilization of RI‐α influences muscle cell differentiation. Identified as a novel interaction partner of MuRF2 and MuRF3 in myocytes, SNX5 functions as a retromer subunit involved in the retrograde transport of endosomes to the TGN [30]. MuRF2 associates with SNX5 and promotes its ubiquitination and UPS‐dependent degradation, whereas MuRF3 counteracts this process, indicating opposing effects on SNX5 stability. Our data also suggest a previously unreported role for MuRF2 and MuRF3 in vesicular trafficking. Because the function of SNX5 in muscle is not well understood, we focused on this protein for further analysis.

MuRF2 and MuRF3 target client proteins for UPS‐dependent degradation [2]. MuRF2 associates with sarcomeric M‐band proteins in mature cardiac myocytes [31] and interacts with titin, MyHC, nebulin, cardiac troponin I and T, myotilin and T‐cap [22, S12]. MuRF3, the least characterized MuRF family member, also interacts with cardiac troponin I [2], though its degradation targets remain unclear.

We report that MuRF2 associates with SNX5 and mediates its ubiquitination and degradation, establishing SNX5 as a novel MuRF2 target. Increasing MuRF2 co‐synthesis reduces SNX5 abundance. Second, coexpression of MuRF2 and SNX5 decreases SNX5 stability, an effect attenuated by the UPS‐inhibitor MG132, confirming UPS dependence. Third, MuRF2‐mediated SNX5‐ubiquitination is RING‐finger dependent. Fourth, MuRF2 mediates ubiquitination of lysine‐290 and lysine‐324 within SNX5, conserved across species. Together, these data indicate that SNX5 is a novel MuRF2‐degradation target. We also found that MuRF3 interacts with SNX5 but does not promote its degradation. This interaction is mediated by MuRF3's coiled‐coil domains and SNX5's BAR domain. The critical role of MuRF3's CC domains align with findings that MuRF proteins require CC domains for protein interactions, including heterodimerization [32]. In addition, the CC domain of MuRF3 is necessary and sufficient for its interaction with glutamylated microtubules that are important for microtubule stabilization [9]. Similarly, SNX5's BAR domain mediates interaction with SNX1, SNX2 [33], FANCA (Fanconi anaemia, complementation group A) [S13], CHC22 (clathrin heavy‐chain 22) [S14] and DOCK180 (an activator of small GTPases) [S15]. Although MuRF3 associates with SNX5, it does not ubiquitinate or degrade it but instead inhibits MuRF2‐dependent SNX5 degradation, suggesting MuRF3 stabilizes SNX5. The mechanism remains unclear but could involve competition for SNX5 binding or MuRF2‐MuRF3 interaction [5], potentially interfering with SNX5 targeting. This represents the first report of an antagonistic MuRF2 and MuRF3 function in myocytes.

Besides these observations, MuRF2 and MuRF3 exhibit structural properties independent of their E3 ligase activity [9]. Both associate with microtubules and stabilize the microtubular network [8, 9, 22], crucial for myogenic differentiation [34] and contractile function [8]. Stable microtubule networks are also essential for intracellular trafficking, with disruptions impairing endosome maturation and retromer‐mediated retrograde transport to the TGN [S16]. Because MuRF2 and MuRF3 localize to microtubules and associate with SNX5‐containing EE, this interaction was of interest.

SNX5 deletion in mice leads to 40% perinatal lethality due to respiratory failure caused by alveolar epithelial dysfunction [S17]. Although SNX5 function in myocytes remains unclear, we found high SNX5 expression in skeletal muscle, heart and myocytes. Our data suggest that MuRF3–SNX5 interaction is important for retromer‐mediated retrograde transport. SNX5 localizes to EE in myocytes, consistent with reports in non‐myocytes [35]. When expressed alone, SNX5 shows a cytosolic and perinuclear pattern, but co‐expression with MuRF3 induces a microtubular staining pattern, suggesting MuRF3 recruits SNX5 to microtubules. Early endosomes align with microtubules, supporting the idea that endosomal trafficking occurs along these structures in myocytes [36]. The partial colocalization of MuRF3 and SNX5 at early endosomes is expected, as retromer‐labelled vesicles undergo dynamic transport along microtubules towards the TGN [S18]. MuRF2 may also be involved in microtubule‐associated vesicular transport, as the MuRF2B isoform interacts with LC3, essential for autophagic vesicle formation [S19], whereas the MuRF2A isoform interacts with p62 and NBR1 in myogenic cells, mediating degradation of ubiquitinated substrates via autophagy [S19]. These findings suggest MuRF2 and MuRF3 contribute to SNX5‐dependent cellular trafficking.

We show that SNX5‐coated vesicles regulate PKA activity, a family of serine–threonine kinases dependent on cAMP. The PKA holoenzyme consists of a regulatory subunit dimer (RI‐α, RI‐β, RII‐α or RII‐β) and two catalytic subunits. We identified RI‐α, the only subunit compensating for excess PKA catalytic activity [20], within SNX5‐coated vesicles. RI‐α physically interacts with SNX5, confirming SNX5‐selective cargo transport in myocytes. PKA inhibits myogenic differentiation [15]. Using CRISPR/Cas9‐generated Snx5‐KO cell lines and siRNA‐mediated SNX5 knockdown, we found that free RI‐α is less stable in SNX5‐deficient myocytes, rescued by proteasome and lysosome inhibition, suggesting degradation via UPS and autophagy. RI‐α ubiquitination and degradation were previously reported in non‐myocytes (HEK cells) [37], consistent with our findings that disrupting SNX5–RI‐α interaction increases RI‐α ubiquitination and reduces stability in myocytes. Free RI‐α localizes to various subcellular compartments, including cytoplasm, nucleus [S20], membranes [38] and multivesicular bodies [20]. We observed that SNX5 mediates uptake of RI‐α to EE where RI‐α undergoes N‐glycosylation, protecting it from UPS‐dependent degradation. PKA regulates myogenic differentiation via two major pathways: the CREB and the HDAC5‐MEF2 axes. PKA‐mediated CREB phosphorylation leads to nuclear translocation and transcription of target genes. Phosphorylated CREB decreases *Ache* (acetylcholinesterase) and increases *Mstn* (myostatin), both inhibitors of myogenic differentiation [25, 28]. Myostatin, a TGF‐family member, suppresses myogenesis [25]. Indeed, SNX5 deletion increased CREB phosphorylation, reduced *Ache* expression and upregulated myostatin, impairing myogenic differentiation. PKA also regulates MEF2 activity via the HDAC‐MEF2 axis [S21]. Class IIa HDACs, including HDAC4 and HDAC5, inhibit MEF2, repressing MEF2‐dependent gene programs [S22]. HDAC5, but not HDAC4, is destabilized in SNX5‐KO myocytes via lysosomal degradation. This aligns with reports that HDAC5, but not HDAC4, controls neurogenic muscle atrophy [S23]. PKA also directly inhibits MEF2, suppressing myogenesis [39]. Increased myostatin expression in SNX5‐KO cells correlates with defective myogenic differentiation. Recombinant myostatin treatment of differentiating C2C12 cells reduced differentiation markers and myoblast fusion, supporting myostatin‐mediated inhibition. Thus, both PKA/CREB and PKA/HDAC5/MEF2D axes contribute to myostatin upregulation and impaired myogenesis.

Our results also align with and extend the findings of Moriscot et al., who demonstrated that MuRF1 and MuRF2 are essential for skeletal muscle regeneration through regulation of the chromatin‐remodelling complex and activation of myogenic gene expression [40]. Moriscot et al. used a mouse model of cardiotoxin‐induced regeneration of the *tibialis anterior* muscle. They showed that MuRF1 and MuRF2 double knockout mice exhibit impaired skeletal muscle regeneration, decreased expression of myogenic regulators such as Myf5, FHL2 and MARP2 and nuclear accumulation of BRG1‐associated factor 57 (BAF57), which is a SWI/SNF chromatin remodelling complex component, indicating a failure in chromatin remodelling. In our study, we identified a novel cytoplasmic mechanism by which MuRF2 and MuRF3 modulate myogenic differentiation. Specifically, we demonstrate that MuRF2 and MuRF3 distinctly modulate the stability of SNX5, which in turn regulates PKA activity and myogenic differentiation via CREB and HDAC5–MEF2 signalling. Together, these findings support a broader regulatory role for MuRF proteins in myogenesis that includes modulation of transcriptional programmes via chromatin remodelling as well as vesicle‐mediated signalling pathways. This implicates that the MuRF family contributes to muscle homeostasis via both nuclear and cytoplasmic mechanisms.

## 6 Limitations

We show that MuRF2 and MuRF3 associate with SNX5, but only MuRF2 promotes ubiquitination and degradation. Whether this results from differential SNX5 binding affinities or MuRF2–MuRF3 heterodimerization remains unclear. Among the MuRF family, MuRF1 is the most extensively studied and is well known for its role in skeletal muscle atrophy. Whether MuRF1 also interacts with SNX5, mediates its ubiquitination or influences SNX5 stability remains unknown. Investigating this would further clarify whether SNX5 regulation is a shared function across MuRF family members or specific to MuRF2 and MuRF3. However, these questions were beyond the scope of the present study and warrant further investigation. Additionally, our data suggest RI‐α is N‐glycosylated in early endosomes, yet its precise subcellular site of glycosylation and necessity for endosomal uptake require further analyses.

## 7 Conclusions

**FIGURE 8:**
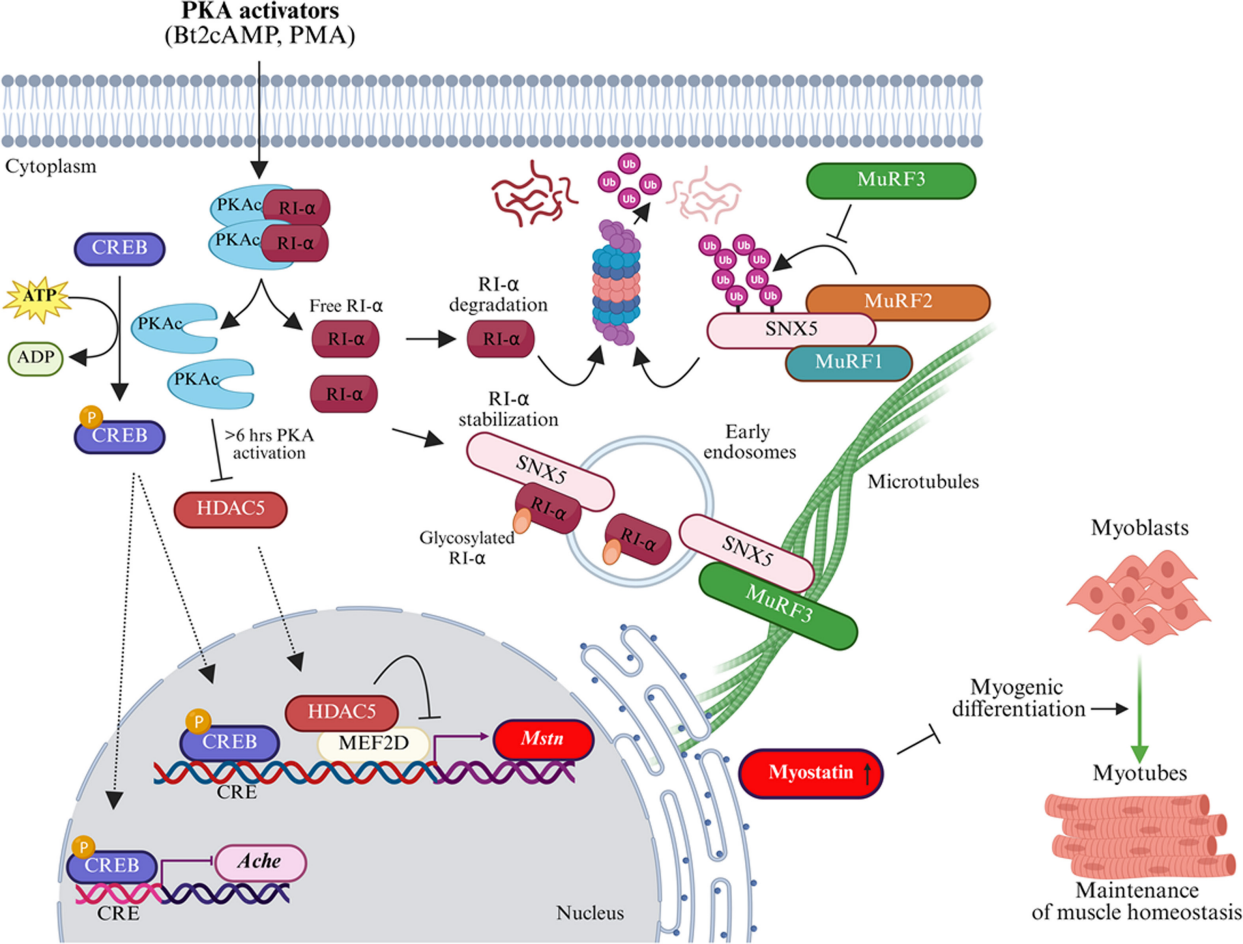
The novel MuRF2 target SNX5 regulates PKA activity through stabilization of RI‐α and controls myogenic differentiation. The working model illustrating the mechanism by which SNX5 contributes to myogenic differentiation in myocytes. MuRF3 interacts with SNX5 at microtubules and reduces MuRF2‐mediated and UPS‐dependent SNX5 degradation. Although MuRF1 also binds to SNX5 (unpublished data), the functional consequences of this interaction are currently unknown. The activation of PKA by Bt2cAMP and PMA results in the dissociation of RI‐α from the PKA tetramer. In the presence of SNX5 free RI‐α interacts with SNX5 and is internalized into SNX5‐coated early endosomes where free RI‐α is stable. Conversely, in the absence of SNX5, free RI‐α degraded through a UPS‐dependent pathway. Once phosphorylated by PKA CREB translocates into the nucleus, binds to CRE sites in the promoter region of *Mstn* and *Ache* that enhances the expression of *Mstn* although it inhibits *Ache* expression. Also, destabilization of HDAC5 by long‐term PKA activation increases MEF2D‐dependent *Mstn* expression. Consequently, elevated myostatin impedes myogenic differentiation disturbing muscle homeostasis.

Our study shows that MuRF2 and MuRF3 play a role in microtubule‐dependent intracellular trafficking of SNX5‐coated early endosomes that are important for the uptake of free RI‐α and for the regulation of PKA activity in myocytes. We identified SNX5 as a novel target for MuRF2‐mediated ubiquitination at lysine‐290 and lysine‐324, leading to its UPS‐dependent degradation. In contrast, although MuRF3 interacts with SNX5, it does not increase SNX5 degradation. We demonstrate that free RI‐α is a novel SNX5‐selective cargo in EE, where it is prevented from UPS‐dependent degradation. The absence of SNX5 leads to an increased UPS‐ and autophagy‐dependent degradation of free RI‐α that causes a higher PKA activity. In response, PKA, through the regulation of MEF2D and CREB, causes an increase in Myostatin and a decrease in acetylcholinesterase expression, which both inhibit myogenic differentiation (Figure 8). The clarification of the regulatory mechanisms of SNX5 and its cargo‐selective functions in muscle development may provide novel therapeutic targets for the treatment of muscle wasting diseases and the promotion of muscle regeneration.

## Ethics Statement

The authors of this manuscript certify that they comply with the ethical guidelines for authorship and publishing in the *Journal of Cachexia, Sarcopenia and Muscle* [41].

## Conflicts of Interest

The authors declare no conflicts of interest.

## Supporting information


**Data S1:** Supporting Information.

## Data Availability

The original contributions presented in the study are included in the article and in the Supporting Information. Further inquiries can be directed to the corresponding author.
