# Supplementary material for: The Novel MuRF2 Target SNX5 Regulates PKA Activity Through Stabilization of RI‐α and Controls Myogenic Differentiation
Source: J Cachexia Sarcopenia Muscle. 2025 Oct 12;16(5):e70103. doi: 10.1002/jcsm.70103 (PMC12515711; doi:10.1002/jcsm.70103)
Supplement: Supplementary file 1 — Data S1: Supporting Information. [file JCSM-16-e70103-s001.pdf]

The novel MuRF2 target SNX5 regulates PKA activity through stabilization of RI-α and controls myogenic differentiation

Ning Li[1,2], Jida Hamati[3], Yi Li[1,3], Björn Brinschwitz[1,2], Mohamed Ghait[1,2], Elisa Martin[1,2], Dörte Lodka[3], Elke Hammer[1,4], Britta Fielitz[1,2], Uwe Völker[1,4], Gunnar Dittmar[5,6], Thomas Sommer[7,8,9], Friedrich C. Luft[3], Jens Fielitz[1,2,3*]

Supplementary Methods

Cell culture experiments

COS-7 cells (ATCC, CRL-1651) and HEK293 cells (ATCC, USA; CRL-1573) were cultured in Dulbecco's Modified Eagle Medium (DMEM, 4.5g/L glucose, Gibco™, Thermo Fisher Scientific, USA) supplemented with 10% fetal bovine serum (FBS), 2 mM glutamine, 1 U/ml penicillin, and 1 µg/ml streptomycin (all from Sigma-Aldrich, Germany) at 37°C in a 5% CO_2 atmosphere. C2C12 cells (ATCC, USA, CRL-1772) were cultivated in growth medium (GM; DMEM, 1g/l glucose, PAN-Biotech, Germany) supplemented with 10% FBS, 2 mM glutamine, 1 U/ml penicillin, and 1 µg/ml streptomycin (all from Sigma-Aldrich, Germany). For differentiation, C2C12 myoblasts at 80% confluency were transferred to differentiation medium (DM; DMEM, 1g/L glucose, 2% FBS, 2 mM glutamine, 1 U/ml penicillin, and 1 µg/ml streptomycin (all from Sigma-Aldrich, Germany) for indicated time points with daily medium exchange. Following chemicals were used to treat cells: cycloheximide (100 µg/ml, CHX), chloroquine (50 µM, CQ), dibutyryl cyclic adenosine monophosphate (1 mM, Bt2cAMP) (all Sigma-Aldrich, Germany), MG132 (C2C12: 10 µM, COS-7: 25 µM, Merck, Germany), bafilomycin A1 (200 nM, BafA1, Cell Signaling, USA), phorbol 12-myristate 13-acetate (100 nM, PMA, InvivoGen, USA), recombinant myostatin (100 ng/ml, MSTN, Proteintech, UK), reconstitution buffer (4 mM HCl, 0.1% BSA).

Generation of cDNA expression plasmids and site directed mutagenesis

The coding sequences of *Trim55* (MuRF2), *Trim54* (MuRF3) and *Snx5* (SNX5) were amplified from mouse muscle cDNA by PCR using primer pairs containing restriction enzyme consensus sequences (primer sequences are shown in Table S1). cDNA expression plasmids were used as a template with specific primer pairs (shown in Table S4) to synthesize deletion mutants. To generate E3 ligase deficient *Trim55* and *Trim63* cDNA expression plasmids cysteine residues Cys42 and Cys50 in MuRF2 and MuRF3 RING-finger domains were mutated

into serine residues using the Phusion™ site-directed mutagenesis kit (Thermo Fisher Scientific, USA). Primers were designed according to the manufacturer's protocol (Table S4). SNX5 lysine-to-arginine mutants were generated by using the QuikChange II site-directed mutagenesis kit (Agilent Technologies, USA) with primers that were designed according to the manufacturer's protocol (Table S4). cDNA expression plasmids were sequence verified.

Transfection

COS-7 and HEK293 cells were transfected using FuGENE-6 (Roche, Switzerland) according to the manufacturer's recommendations. Lipofectamine and PLUS™ reagent (both Invitrogen, USA) were used to transfect cDNA expression plasmids into C2C12 cells according to the manufacturer's protocol. For siRNA transfection, non-target (NT)-siRNA (D-001810-10-05) and SNX5-siRNA (J-060939-09-1101) were transfected into C2C12 cells with Dharmafect3 (all from Dharmacon, USA) according to the manufacturer's protocol.

Retrovirus production and transduction of C2C12 cells

The coding sequence of *Snx5* were PCR amplified (Primers are shown in Table S5) and subcloned into a retroviral expression plasmid (pMP71-IRES-GFP). Plat-E cells (Platinum-E, Cell Biolabs, USA) that were used for retrovirus generation, were cultured in Eagle's Minimum Essential (EME) medium (4.5 g/l glucose, 10 % FBS, 1% penicillin and streptomycin, 1 µg/ml puromycin, 10 µg/ml blasticidin S (all from Sigma-Aldrich, Germany)) in a humidified 5% CO_2 atmosphere at 37°C. 16 µg of the retroviral constructs were transfected into Plat-E cells with 48 µg of polyethylenimine MAX (PEI "MAX", Polysciences, USA) according to the manufacturer's protocol. Retrovirus was isolated from cell culture supernatants 48 hours after transfection. Retrovirus was transduced into C2C12 cells using 5 µg/ml polybrene (Santa Cruz, USA) and spinoculation at 800xg for 90 min at 32°C.

RNA isolation, cDNA synthesis and quantitative real-time PCR

Total RNA was isolated from skeletal muscle or cultured cells using TRIzol® Reagent

(Invitrogen, USA) according to the manufacturer's protocol. cDNA synthesis of 1 µg of RNA per sample was carried out by using the SuperScript® First-Strand Synthesis System (Invitrogen, USA). Quantitative real-time polymerase chain reaction (qRT-PCR) was performed using Power SYBR® Green PCR Master Mix (Applied Biosystems, USA) and self-designed primers (for primer sequences see Table S6), in a Step-One Plus or a QuantStudio 3 thermocycler (both Applied Biosystems, USA) as described recently using a cDNA standard curve [1, 2]. Gene expression was normalized to the stably expressed glyceraldehyde-3-phosphate dehydrogenase (*Gapdh*).

Immunostaining of myoblasts and myotubes in vitro

For immunofluorescence microscopy cells were cultured in IBIDI slides (Ibidi GmbH, Germany), fixed with 4% paraformaldehyde (pH 7.0; 15 min, room temperature), permeabilized with 0.2% Triton X-100 in PBS for 30 min at room temperature, blocked with 5% goat serum (Dako, Germany) for 1 hour at room temperature corresponding to the primary antibodies host and incubated with specific primary antibody overnight at 4°C. After washing with PBS containing 0.2% Tween20 (Carl Roth, Germany), cells were incubated with fluorescent secondary antibody for 1 hour at room temperature in a dark environment. Stained cells were embedded in ProLong Gold® Antifade Reagent that contained DAPI for nuclei staining (Invitrogen, USA). Pictures were taken with a Keyence microscope (BZ-X810, Keyence, Japan) and the Zeiss confocal laser scanning microscope (LSM 980 with Airyscan 2, Carl Zeiss Inc., Germany), and analyzed with BZ-X800 Analyzer (Keyence, Japan) and analysis software (Zen v. 3.3.89, Carl Zeiss Inc., Germany), respectively. Myogenic differentiation index was calculated as the percentage of nuclei in fast MyHC positive myotubes related to the total number of nuclei per field of view. The fusion index was calculated as the percentage of nuclei number in fast MyHC positive myotubes.

Protein extraction and immunoblotting

Protein analyses were performed as recently published [1]. Shortly, cells were lysed in radioimmunoprecipitation assay (RIPA) buffer (50 mM Tris HCl, pH 7.5, 150 mM NaCl, 0.5% (w/v) sodium-deoxycholate, 1% (v/v) nonident P-40, 1 mM EDTA (ethylenediaminetetraacetic acid), 0.2% (w/v) sodium dodecyl sulfate (SDS)) containing protease (cOmplete, Roche, Germany) and phosphatase (1 mM Na_3VO_4, 1 mM phenylmethylsulfonyl fluoride, PhosSTOP (Roche, Germany)) inhibitors at 4°C. Lysates were cleared by centrifugation (4°C, 10 min, 12,000xg). The supernatant was assayed for protein concentration using the Bradford protein assay (Bio-Rad Laboratories, USA), frozen and stored at -80°C until usage. Proteins were separated by 10% sodium dodecyl sulfate–polyacrylamide gel electrophoresis (SDS-PAGE) and blotted onto nitrocellulose membranes (NC; Amersham Pharmacia Biotech, UK). Membranes were blocked with 5% BSA or 5% dry milk in Tris-buffered saline with 0.1% Tween20 (TBST), then incubated with primary and secondary antibodies as indicated and the signal was visualized with the ChemiDoc™ MP Imaging System (Bio-Rad Laboratories, USA). The ImageLab™ 6.0.1 software (Bio-Rad Laboratories, USA) was used for image analysis. *Protein sample preparation for affinity purification followed by MS analysis:* Cells were grown in 6-well plates or T75 cell culture flasks, and harvested using lysis buffer (10 mM TRIS, 150 mM NaCl, 100 ng/ml cOmplete). Following three freeze&thaw cycles cell membranes were destroyed using a syringe (needle size Ø 0.40 x 20 mm). The supernatant was precleared by low speed centrifugation (10 min, 300xg); resulting supernatants were subjected to high speed centrifugation (20 min, 50,000xg). The clear middle phase was collected for Nickel-NTA (Thermo Fisher Scientific, USA) pull-down and subsequent MS analysis. *Protein sample preparation for mass spectrometry analysis of SNX5-enriched endosomes:* Cell membranes were destroyed using a syringe (needle size Ø 0.40 x 20 mm) in homogenization buffer (250 mM sucrose, 3 mM imidazole, 1 mM EDTA, 0.03 mM cycloheximide, cOmplete and phosphatase inhibitor cocktail 1 and 2) to isolate the post-nuclear supernatant (PNS), in which

endosomes are maintained. The SNX5-coated endosomes were purified from PNS using Pierce™ Protein A/G Agarose (Thermo Fisher Scientific, USA) that was pre-coated with anti-SNX5 antibody overnight at 4°C. Subsequently, SNX5-precipitates were subjected to MS analysis or to other indicated assays. *For nucleus and cytoplasm fractionation:* All preparations were performed on ice. Cytoplasm fractions were obtained from the PNS as indicated. Next, nuclear proteins were isolated in NE-PER™ Nuclear Extraction Reagents (Thermo Fisher Scientific, USA). Nuclei pellets separated from the PNS isolation were resuspended in nuclear lysis buffer for 30 min, 4°C and cleared by centrifugation (4°C, 10 min, 12,000xg).

Affinity purification and coimmunoprecipitation

Proteins were isolated from transfected cells using lysis buffer (50 mM potassium-phosphate buffer (KH_2PO_4 (pH 4.0)/K_2HPO_4 (pH 9.3)), 150 mM NaCl, cOmplete, 0.2% TritonX-100, pH7.4). 10% of cell lysates were used for input controls. Nickel-NTA and anti-FLAG M2 Affinity Gel (Sigma-Aldrich, Germany) were used to precipitate His- and FLAG-tagged proteins, respectively. Proteins from input control and precipitates were subjected to Western blot analysis with the indicated antibodies. *Sample preparation for proteinase K and PNGase F digestion:* The SNX5-coated endosomes were purified from PNS using Pierce™ Protein A/G Agarose that was pre-coated with anti-SNX5 antibody overnight at 4°C. SNX5-precipitates were subjected to proteinase K (New England Biolabs, Germany) digestion. Afterwards, proteinase K was inactivated by 1 hour of PMSF (Sigma-Aldrich, Germany) treatment. SNX5-precipitates were subjected to PNGase F (New England Biolabs, Germany) as indicated referring to the manufacturer’s protocol.

Cycloheximide chase and Ubiquitination assays

Protein stability assays were performed by exposing transfected cells to cycloheximide (CHX, 100 µg/ml) to inhibit protein synthesis. Proteins were isolated at indicated time points and analyzed by Western blot analyses with indicated antibodies. All CHX experiments were

performed independently and at least twice using biological duplicates each. Representative Western blots and their densitometric analyses are shown.

In vitro ubiquitination assays were performed as recently published [1]. Briefly, COS-7 cells were transfected as indicated for 48 hours. Prior to harvesting, cells were treated with either vehicle (0.25% DMSO) or the proteasome inhibitor MG132 (25 μM) for 6 hours. Cells were lysed in lysis buffer (50 mM potassium-phosphate buffer (KH_2PO_4 (pH 4.0)/K_2HPO_4 (pH 9.3)), 150 mM NaCl, cOmplete, 25mM N-Ethylmaleimide (NEM, Sigma-Aldrich, Germany), 0.2% TritonX-100, pH7.4) and 10% of the lysates were used for input controls. Lysates were immunoprecipitated (IP) with anti-FLAG M2 affinity gel overnight at 4°C. Proteins from input control and precipitates were subjected to Western blot analyses with the indicated antibodies.

Cell fractionation and PKA assays

Sucrose gradient (30%, 40%, 50%, 60% sucrose) fractionation was performed from 400 μl PNS by ultracentrifugation at 130,000xg for 3 hours at 4°C. Nine fractions were collected for Western blot analyses with indicated antibodies. Endosome enriched fractions were isolated from PNS by ultracentrifugation at 120,000xg for 1 hour at 4°C. The supernatant was collected as the cytosol fraction. The membrane-containing pellet was washed with ice cold PBS and resuspended in RIPA buffer. Proteins from the isolated fractions were analyzed by Western blot analyses with indicated antibodies.

PKA activity was measured using the PKA Colorimetric Activity Kit (Thermo Fisher Scientific, USA) according to the manufacturer's instruction.

Cross-linking experiments

Cross-linking experiments were performed according to a previously published protocol [3]. Briefly, after treatment, cells were harvested in lysis buffer (20mM, pH7.5 HEPES-KOH, 10 mM KCl, 1.5 mM $MgCl_2$, 1 mM EDTA, 1 mM EGTA (ethylene glycol-bis(β-aminoethyl ether)-N,N,N',N'-tetraacetic acid), and 320 mM sucrose). Following centrifugation, the

supernatant and the pellet were diluted in CHAPS ((3-[(3-cholamidopropyl) dimethylammonio]-1-propanesulfonate)) buffer (20mM, pH7.5 HEPES-KOH, 5 mM $MgCl_2$, 0.5 mM EGTA, 0.1 mM PMSF and 0.1% CHAPS) and then incubated with non-cleavable disuccinimidyl suberate (DSS, 4 mM, Thermo Fisher Scientific, USA) for 30 min at 4°C. After resuspension in Laemmli buffer (Bio-Rad Laboratories, USA), proteins were analyzed by immunoblotting.

ChIP-qRT-PCR

Chromatin immunoprecipitation (ChIP) assay was performed according to the manufacturer's protocol using the CUT&RUN Assay Kit (Cell Signaling, USA). Briefly, fixed C2C12 cells were incubated with concanavalin A-coated magnetic beads and permeabilization was performed with digitonin (0.05%)-containing buffer. Afterwards, cells were incubated with anti-MEF2D antibody (1 μg) or anti-tri-methyl-histone H3 (Lys4) (clone C42D8) antibody (0.5 μg) and rabbit IgG antibody (negative control for anti-MEF2D, 1 μg) or rabbit isotype specific IgG antibody (negative control for anti-Histone H3, 0.5 μg) overnight at 4°C. The cell-bead-antibody slurry was incubated with protein A/G-MNase (Protein A and Protein G IgG binding domains fused to micrococcal nuclease) for 1 hour at 4°C. CUT&RUN fragments were released by incubation for 10 min at 37°C followed by centrifugation for 5 min at 16,000xg. Reverse crosslinking of released CUT&RUN fragments was performed in 10% SDS and proteinase K (20mg/ml) at 65°C overnight. An input sample was digested with proteinase K (20 mg/ml) and RNase A at 55°C for 1 hour. Afterwards, the input sample was sonicated (Bioruptor Pico, Diagenode, Belgium) for 35 cycles (30sec ON/ 30 seconds OFF) at 4°C. qPCR with self-generated primers for MEF2D and company provided primers for Histone H3 (Table S4) was used to determine changes in DNA amounts.

Antibodies

Following antibodies were used: mouse anti-GAPDH (clone 6C5), rabbit anti-Myc

(both from Merck, Germany), rabbit anti-FLAG, mouse anti-HA, rabbit anti-RI-α, rabbit anti-PKA catalytic subunit, rabbit anti-phospho-CREB (Ser133), rabbit anti-CREB (48H2), rabbit anti-HDAC4, anti-mouse IgG HRP linked, anti-rabbit IgG HRP linked (all from Cell Signaling, USA), mouse anti-Myc, mouse anti-myosin (slow, NOQ7), mouse anti-myosin (fast, My32), mouse anti-myogenin (all from Sigma-Aldrich, Germany), rabbit anti-SNX5, rabbit anti-Myostatin (GDF8), rabbit anti-RAB5 (RAB5A) (all from Proteintech, UK), rabbit anti-LaminB1, rabbit anti-Histone H3 (both from Abcam, UK), mouse anti-MEF2D, mouse anti-EEA1, mouse anti-LAMP1 (all from BD Biosciences, USA), rabbit anti-MuRF2, rabbit anti-MuRF3 (own production[2]), anti-mouse Alexa Fluor®488, anti-rabbit Alexa Fluor®488, anti-mouse Alexa Fluor®555, anti-rabbit Alexa Fluor®555, and anti-mouse Alexa Fluor®647 (all from Life Technologies, USA).

Mass spectrometric analysis

Determination of MuRF3 interaction partners: precipitates containing MuRF3 and protein-interaction partners were resuspended in 100 µl denaturation buffer (10mM HEPES, pH 8.0, 6M urea, 2M thiourea). Disulfide bonds were reduced with 10 mM tris(2-carboxyethyl) phosphine (TCEP) and cysteine groups were alkylated with 55 mM 2-chloroacetamide (CAA). The proteins were digested with Lys-C overnight. Peptides were extracted, desalted and stored on reversed-phase (C18) StageTips [4]. The SILAC labelled IP pairs were mixed prior to the MS analysis. *High throughput LC-MS/MS analysis:* After Stage-Tip extraction, the eluted peptides were lyophilized and resuspended in 3% trifluoroacetic acid/5% acetonitrile. Peptides were separated on a Proxeon nLC-II system (Thermo Fisher Scientific, USA), resolved with a reversed-phase column (Dr. Maisch GmbH C18) by a gradient from 4 to 42% acetonitrile in 240 min. MS and MS/MS spectra were recorded on a QExactive mass spectrometer (Thermo Fisher Scientific, USA). The mass spectrometer was operated in a data-dependent acquisition mode with dynamic exclusion enabled (30s). Survey scans (mass range 300-1500 Th) were

acquired at a resolution of 70,000, with the twenty most abundant multiply charged ($z \geqslant 2$) ions selected with a 3-Th isolation window for HCD fragmentation. MS/MS scans were acquired at a resolution of 35,000 and injection time of 120ms, ACG 10e5. Processing of mass spectrometry data: protein and peptide quantitation information were extracted from MaxQuant 1.2.2.5 [5]. Results were filtered to 1% false discovery rate (FDR) at peptide and protein level by MaxQuant. Variable modifications were set to oxidation of methionines and fixed modifications were set to carbamidomethylation of cysteines. The SILAC parameters were set to light and heavy labelling on lysine (+8Da). Additionally, the match between runs and the requantify option were activated. Further analysis was performed using the R-statistical language (www.R-project.org). *Determination of proteins contained in SNX5 precipitates:* Sepharose with precipitated SNX5 and interacting proteins were reconstituted in 30 µl Tris-HCl buffer (pH 8.0, 50 mM) containing 10 mM dithiotreitol (DTT) and 2% SDS. Samples were heated at 95°C for 5 min and the protein containing supernatant was collected after centrifugation (5 min, 13,800xg). Ten µl sample was subjected to a bead based SP3 protocol applied for protein digestion and peptide purification [6]. The resulting peptides were separated by Liquid chromatography (LC; Ultimate 3000, Thermo Electron, Germany) before data-dependent acquisition of MS data on a Q Exactive Plus mass spectrometer (Thermo Electron, Germany). MS data were analyzed in Proteome discoverer 2.3 (Thermo Electron, Germany). Cysteine carbamidomethylation was set as static modification, oxidation at methionine and acetylation at protein N-terminus were defined as variable modifications, and up to two missed cleavages were allowed. Proteins were only considered for further analyses, when identified by at least one unique peptide (FDR<0.05). Further details are provided in Table S7. The mass spectrometry proteomics data have been deposited to the ProteomeXchange Consortium via the PRIDE [7] partner repository with the dataset identifiers PXD058900 (MuRF3 interaction partners) and PXD057619 (proteins contained in SNX5 precipitates).

References for Supplementary Methods section

1. Li Y, Dormann N, Brinschwitz B, Kny M, Martin E, Bartels K, et al. SPSB1-mediated inhibition of TGF-beta receptor-II impairs myogenesis in inflammation. J Cachexia Sarcopenia Muscle. 2023;14:1721-36.

2. Lodka D, Pahuja A, Geers-Knorr C, Scheibe RJ, Nowak M, Hamati J, et al. Muscle RING-finger 2 and 3 maintain striated-muscle structure and function. J Cachexia Sarcopenia Muscle. 2016;7:165-80.

3. Fernandes-Alnemri T, Wu J, Yu JW, Datta P, Miller B, Jankowski W, et al. The pyroptosome: a supramolecular assembly of ASC dimers mediating inflammatory cell death via caspase-1 activation. Cell Death Differ. 2007;14:1590-604.

4. Rappsilber J, Mann M, Ishihama Y. Protocol for micro-purification, enrichment, pre-fractionation and storage of peptides for proteomics using StageTips. Nat Protoc. 2007;2:1896-906.

5. Cox J, Mann M. MaxQuant enables high peptide identification rates, individualized p.p.b.-range mass accuracies and proteome-wide protein quantification. Nat Biotechnol. 2008;26:1367-72.

6. Sielaff M, Kuharev J, Bohn T, Hahlbrock J, Bopp T, Tenzer S, et al. Evaluation of FASP, SP3, and iST Protocols for Proteomic Sample Preparation in the Low Microgram Range. J Proteome Res. 2017;16:4060-72.

7. Perez-Riverol Y, Bai J, Bandla C, Garcia-Seisdedos D, Hewapathirana S, Kamatchinathan S, et al. The PRIDE database resources in 2022: a hub for mass spectrometry-based proteomics evidences. Nucleic Acids Res. 2022;50:D543-D52.

Supplementary References

S1. Lange S, Xiang F, Yakovenko A, Vihola A, Hackman P, Rostkova E, et al. The kinase domain of titin controls muscle gene expression and protein turnover. Science. 2005;308:1599-

603.

S2. Witt CC, Witt SH, Lerche S, Labeit D, Back W, Labeit S. Cooperative control of striated muscle mass and metabolism by MuRF1 and MuRF2. EMBO J. 2008;27:350-60.

S3. Willis MS, Wadosky KM, Rodriguez JE, Schisler JC, Lockyer P, Hilliard EG, et al. Muscle ring finger 1 and muscle ring finger 2 are necessary but functionally redundant during developmental cardiac growth and regulate E2F1-mediated gene expression in vivo. Cell Biochem Funct. 2014;32:39-50.

S4. Bodine SC, Latres E, Baumhueter S, Lai VK, Nunez L, Clarke BA, et al. Identification of ubiquitin ligases required for skeletal muscle atrophy. Science. 2001;294:1704-8.

S5. Ong SE, Blagoev B, Kratchmarova I, Kristensen DB, Steen H, Pandey A, et al. Stable isotope labeling by amino acids in cell culture, SILAC, as a simple and accurate approach to expression proteomics. Mol Cell Proteomics. 2002;1:376-86.

S6. Mann M. Functional and quantitative proteomics using SILAC. Nat Rev Mol Cell Biol. 2006;7:952-8.

S7. Kvainickas A, Jimenez-Orgaz A, Nagele H, Hu Z, Dengjel J, Steinberg F. Cargo-selective SNX-BAR proteins mediate retromer trimer independent retrograde transport. J Cell Biol. 2017;216:3677-93.

S8. Pan T, Gao S, Cui X, Wang L, Yan S. APC/CCDC20 targets SCFFBL17 to activate replication stress responses in Arabidopsis. Plant Cell. 2023;35:910-23.

S9. Da Graca J, Morel E. Canonical and Non-Canonical Roles of SNX1 and SNX2 in Endosomal Membrane Dynamics. Contact (Thousand Oaks). 2023;6:25152564231217867.

S10. Kwon SH, Oh S, Nacke M, Mostov KE, Lipschutz JH. Adaptor Protein CD2AP and L-type Lectin LMAN2 Regulate Exosome Cargo Protein Trafficking through the Golgi Complex. J Biol Chem. 2016;291:25462-75.

S11. Grade CVC, Mantovani CS, Alvares LE. Myostatin gene promoter: structure, conservation and importance as a target for muscle modulation. J Anim Sci Biotechnol. 2019;10:32.

S12. Moriscot AS, Baptista IL, Bogomolovas J, Witt C, Hirner S, Granzier H, et al. MuRF1 is a muscle fiber-type II associated factor and together with MuRF2 regulates type-II fiber trophicity and maintenance. J Struct Biol. 2010;170:344-53.

S13. Otsuki T, Kajigaya S, Ozawa K, Liu JM. SNX5, a new member of the sorting nexin family, binds to the Fanconi anemia complementation group A protein. Biochem Biophys Res Commun. 1999;265:630-5.

S14. Greig J, Bates GT, Yin DI, Briant K, Simonetti B, Cullen PJ, et al. CHC22 clathrin recruitment to the early secretory pathway requires two-site interaction with SNX5 and p115. EMBO J. 2024 Oct;43(19):4298-4323.

S15. Hara S, Kiyokawa E, Iemura S, Natsume T, Wassmer T, Cullen PJ, et al. The DHR1 domain of DOCK180 binds to SNX5 and regulates cation-independent mannose 6-phosphate receptor transport. Mol Biol Cell. 2008;19:3823-35.

S16. Ghasemizadeh A, Christin E, Guiraud A, Couturier N, Abitbol M, Risson V, et al. MACF1 controls skeletal muscle function through the microtubule-dependent localization of extra-synaptic myonuclei and mitochondria biogenesis. Elife. 2021;10:e70490.

S17. Im SK, Jeong H, Jeong HW, Kim KT, Hwang D, Ikegami M, et al. Disruption of sorting nexin 5 causes respiratory failure associated with undifferentiated alveolar epithelial type I cells in mice. PLoS One. 2013;8:e58511.

S18. Hierro A, Gershlick DC, Rojas AL, Bonifacino JS. Formation of Tubulovesicular Carriers from Endosomes and Their Fusion to the trans-Golgi Network. Int Rev Cell Mol Biol. 2015;318:159-202.

S19. Pizon V, Rybina S, Gerbal F, Delort F, Vicart P, Baldacci G, et al. MURF2B, a novel LC3-binding protein, participates with MURF2A in the switch between autophagy and ubiquitin proteasome system during differentiation of C2C12 muscle cells. PLoS One. 2013;8:e76140.

S20. Gupte RS, Weng Y, Liu L, Lee MY. The second subunit of the replication factor C

complex (RFC40) and the regulatory subunit (RIalpha) of protein kinase A form a protein complex promoting cell survival. Cell Cycle. 2005;4:323-9.

S21. Backs J, Worst BC, Lehmann LH, Patrick DM, Jebessa Z, Kreusser MM, et al. Selective repression of MEF2 activity by PKA-dependent proteolysis of HDAC4. J Cell Biol. 2011;195:403-15.

S22. Lu J, McKinsey TA, Zhang CL, Olson EN. Regulation of skeletal myogenesis by association of the MEF2 transcription factor with class II histone deacetylases. Mol Cell. 2000;6:233-44.

S23. He T, Huang J, Chen L, Han G, Stanmore D, Krebs-Haupenthal J, et al. Cyclic AMP represses pathological MEF2 activation by myocyte-specific hypo-phosphorylation of HDAC5. J Mol Cell Cardiol. 2020;145:88-98.

Supplementary Tables

Table S1. Primers crRNA targeting SNX5 and for generation of cDNA expression plasmids.

Primer Name (restriction site)	Oligonucleotide sequence (5'-3')
SNX5-targeted crRNA	ACUGAAACAACGGAU
MuRF1 Flag for (EcoRI)	GCGAATTCGATTATAAATCTAGCCTGA
MuRF1 Flag rev (ApaI)	GCGGGCCCTCATTGGTGTTCTTCTTT
MuRF2 Flag for (ClaI)	GCATCGATAGCACTTCTCTGAATTACAAGTCTT
MuRF2 Flag rev (ApaI)	GCGGGCCCTTATTCATTTAGGGAATT
MuRF3 Flag for (EcoRI)	GCGAATTCAACTTCACGGTGGGTTTCAA
MuRF3 Flag rev (ApaI)	GCGGGCCCTCAGTGCAGGCCTGAGCCTTC
SNX5 His/Myc for (BamHI)	CGGGATCCATGGCCGCGGTTCCCGAGTT
SNX5 His/Myc rev (KpnI)	GGGGTACCGTTGTTCTTGAATAAGTCGATGCAGCTC

SNX5 Flag for (ClaI)	CCATCGATGCCGCGGTTCCCGA
SNX5 Flag rev (XbaI)	GCTCTAGATCAGTTGTTCTTGAATAAGTCGATGC
MuRF1 His/Myc for (EcoRI)	CAGAATTCATGGATTATAAATCTAGCCTG
MuRF1 His/Myc rev (KpnI)	CTTGGTACCTTGGTGTTCTTCTTTACCCTC
MuRF2 His/Myc for (XhoI)	GACTCGAGATGAGCACTTCTCTGAATTAC
MuRF2 His/Myc rev (KpnI)	CTTGGTACCTTCATTTAGGGAATTCAACCAG
MuRF3 His/Myc for (XbaI)	TCTAGACTATGAACTTCACGGTGGGTTTCAA
MuRF3 His/Myc rev (KpnI)	GGTACCGTGCAGGCCTGAGCCTTCTGGCAC

Protein IDs	Protein Names	Gene Names	Uniprot	Ratio M/L Normalized MSC04526	Ratio M/L Normalized MSC04526 Significance B
IPI00457401;I	Transmembrane BAX inhibitor motif-containing	Tmbim6	Q9D2C7;E0CX98;E0CXR0;	7,330	1,646E-16
IPI00652882;I	Protein fat-free homolog	Ffr	Q3UVL4-1;Q3UVL4;Q3UVL	4,572	4,139E-07
IPI00830533;I	Tropomyosin-1	Tpm1	E9Q454;P58771-1;P58771	4,541	3,006E-09
IPI00331708;I	Myocardin-related transcription factor A	Mkl1	Q8K4J6-1;Q8K4J6;Q3U1I6	4,108	5,991E-06
IPI00406306;I	Tumor suppressor p53	P53	P02340;O70366;Q549C9;(	3,586	8,696E-05
IPI00120617;I	AN1-type zinc finger protein 2A	Airap	Q9JII7;D3YUL0	3,425	5,317E-08
IPI00125454;I	DnaJ homolog subfamily A member 4	Dnaja4	Q9JMC3;Q8R1X2	3,402	6,639E-08
IPI00125140	Activity-regulated cytoskeleton-associated prote	Arc	Q9WV31	3,053	9,348E-04
IPI00308785;I	Cyclooxygenase-2	Cox2	Q05769;Q3UMR6;Q543K3	2,978	1,268E-03
IPI00346073;I	Heat shock 70 kDa protein 1	Hsp70a1	P17879;A1E2B8;Q61698	2,978	3,668E-05
IPI00989436;I	HCV NS5A-transactivated protein 9 homolog	Ns5atp9	Q9CQX4	2,873	1,925E-03
IPI00138274;I	Alpha(B)-crystallin	Crya2	P23927;Q52L78;E9QMA0	2,867	6,884E-06
IPI00128522;I	Heat shock 25 kDa protein	Hsp25	P14602-1;P14602;Q545F4	2,866	6,960E-06
IPI00113117	CCN family member 1	Ccn1	P18406;Q3TX21	2,848	8,171E-05
IPI00322594	CCN family member 2	Ccn2	P29268;Q91V29	2,825	9,599E-06
IPI00626662;I	Aldehyde dehydrogenase family 1 member A1	Ahd2	P24549	2,738	1,878E-05
IPI00331564;I	Dihydrolipoyl dehydrogenase	Dld	Q3TIE8;O08749	2,730	3,319E-03
IPI00108058	CDK-interacting protein 1	Cdkn1a	P39689;Q4FK34;Q564P6	2,701	1,947E-04
IPI00132942	Nuclear distribution protein C homolog	Nudc	O35685;A2A9F5	2,615	8,901E-04
IPI00987945;I	SRY-box containing gene 9	Sox9	Q571J2;Q04887;B1AVH1;(	2,592	5,525E-05
IPI00136253;I	DnaJ (Hsp40) homolog, subfamily B, member 1	Dnajb1	Q9QYJ3;Q3TIT6;Q3TU79;(	2,591	3,624E-04
IPI00515257;I	RNA polymerase B transcription factor 3	Btf3	Q64152-1;Q64152;Q6415	2,574	3,965E-04
IPI00124640	Acrogranin	Grn	P28798;Q3TVQ3;Q3TW77;	2,572	5,884E-03
IPI00117689;I	Cavin-1	Ptrf	O54724	2,547	4,601E-04
IPI00120984	NADH dehydrogenase [ubiquinone] 1 alpha subu	Ndufa8	Q9DCJ5	2,537	6,646E-03
IPI00132958	Acyl-coenzyme A thioesterase 13	Acot13	Q9CQR4;Q4VA32	2,530	1,331E-03
IPI00131577;I	Heme oxygenase 1	Hmox1	P14901;Q3U5H8;Q3U5U6;	2,517	5,421E-04
IPI00132539	Basic transcription factor 3-like 4	Btf3l4	Q9CQH7;A2A7Z4;Q78IG7	2,492	1,595E-03
IPI00110370;I	Tissue inhibitor of metalloproteinases 3	Timp3	P39876;Q54AE5;Q6GXA7	2,487	1,161E-04
IPI00131620;I	DNA polymerase kappa	Dinb1	Q9QUG2-1;Q9QUG2;Q5Q	2,433	9,485E-03
IPI00110760	DnaJ homolog subfamily B member 4	Dnajb4	Q9D832	2,403	2,393E-03
IPI00453954	Transcription elongation factor A protein-like 8	Tceal8	Q9CZY2	2,366	2,815E-03
IPI00421223	Tropomyosin-4	Tpm4	Q6IRU2	2,363	2,855E-03
IPI00752148;I	C9orf119 homolog	2900010J23Rik	Q8K3D3-2;Q8K3D3;Q8K3I	2,351	2,899E-04
IPI00111793;I	Entactin	Ent	P10493;Q3TKX9	2,326	1,350E-02
IPI00119202;I	Calgizzarin	S100a11	P50543	2,193	2,802E-03
IPI00399972;I	Transforming acidic coiled-coil-containing prote	Tacc1	Q6Y685-1;Q6Y685;Q6Y68	2,172	2,183E-02
IPI00112347	Inhibin beta A chain	Inhba	Q04998;Q3UXL8;Q3UY39;	2,156	6,891E-03
IPI00320462	Biphenyl hydrolase-like protein	Bphl	Q8R164;Q3TDN8	2,153	2,317E-02
IPI00263863;I	Heat shock protein 1 (Chaperonin 10)	Hspe1	Q64433;Q4KL76;Q9JI95	2,086	9,122E-03
IPI00678003;I	Inosine triphosphate pyrophosphatase	Itpa	Q9D892;Q60I30;Q3U589	2,051	3,134E-02
IPI00130225	Sorting nexin-4	Snx4	Q91YJ2;Q80X54	2,035	1,112E-02
IPI00173343	Oligoribonuclease, mitochondrial	Rexo2	Q9D8S4;Q3T9B4;Q3TAV0;	2,035	1,113E-02
IPI00403336;I	Optic atrophy protein 1	Opa1	P58281-2;P58281;Q8BK9	2,029	2,075E-03
IPI00125899;I	Beta-catenin	Ctnnb1	Q02248;Q3UZT7;Q80VE7;	2,021	1,172E-02
IPI00170101	Optineurin	Optn	Q8K3K8	2,018	3,444E-02
IPI00228343	Ferrochelatase, mitochondrial	Fech	P22315;Q3UC49;Q544X6;	1,982	2,703E-03
IPI00132903	Ubiquitin-fold modifier-conjugating enzyme 1	Ufc1	Q9CR09	1,947	1,556E-02
IPI00625105;I	U1 small nuclear ribonucleoprotein 70 kDa	Snrnp70	Q62376-1;Q62376;A2RS6	1,928	4,433E-02
IPI00132208;I	DnaJ homolog subfamily A member 1	Dnaja1	P63037;Q3TK61;Q5NTY0;I	1,907	1,005E-02
IPI00316740;I	Damage-specific DNA-binding protein 1	Ddb1	Q3U1J4;Q91YC8;Q3ULS8	1,897	4,828E-02
IPI00112053	Sorting nexin-5	Snx5	Q9D8U8;A2ANA4;Q3TJN6;	1,870	2,062E-02
IPI00830178;I	Angiomotin	Amot	Q8VHG2-1;Q8VHG2;Q8VH	1,846	2,247E-02
IPI00121427	S100 calcium-binding protein A6	S100a6	P14069;Q545I9	1,845	1,300E-02
IPI00606184;I	Zinc finger protein 703	Znf703	P0CL69	1,838	2,305E-02
IPI00331318;I	Palmitoyl-protein hydrolase 1	Ppt1	O88531;Q3TAR8;Q3U6J9;(	1,835	5,928E-03
IPI00322530;I	Acyl-CoA desaturase 1	Scd1	P13516;Q3UXG5;Q547C4;	1,823	2,433E-02
IPI00469329;I	3-hydroxy-3-methylglutaryl-coenzyme A reducta	Hmgcr	Q01237;Q6PB59;Q8BV96;	1,806	2,586E-02
IPI00675897	Muscle-specific RING finger protein 2	Trim55	Q8C6Y1	1,804	2,602E-02
IPI00381563;I	Cytospin-B	Cytsb	Q5SXY1-3;Q5SXY1;Q5SXY	1,799	2,647E-02
IPI00850843;I	AHNAK Nucleoprotein 2	Ahnak2	E9PYB0;Q3UUE0	1,754	3,096E-02
IPI00114209;I	Glutamate dehydrogenase 1, mitochondrial	Glud1	P26443;Q3TSQ7	1,732	2,034E-02
IPI00124751;I	Transient receptor potential cation channel subf	Trpc4ap	Q9JLV2-1;Q9JLV2;Q3TB80	1,692	3,805E-02
IPI00310323;I	Retinoid-inducible serine carboxypeptidase	Scpep1	Q920A5;Q9D625;Q99J29	1,686	3,882E-02
IPI00111218;I	Aldehyde dehydrogenase 2, mitochondrial, isofo	Aldh2	P47738;Q3TVM2;Q3U6I3;(	1,658	2,685E-02
IPI00338785;I	Laminin B1	Lamb-1	P02469;B9EKB0;Q9QN70;(	1,645	4,438E-02
IPI00830443;I	Caseinolytic peptidase B protein homolog	Clpb	E9PY58;Q3TXD4;Q3U3U6;	1,636	1,571E-02
IPI00230476;I	Diaphanous-related formin-3	Diap3	Q9Z207;Q3TSX1;Q3UU77;	1,631	1,609E-02
IPI00776049;I	Translation initiation factor IF-2	Mtif2	Q5M6W6;Q91YJ5;Q5M6W	1,609	4,977E-02
IPI00453812	CTTNBP2 N-terminal-like protein	Cttnbp2nl	Q99LJ0;Q922L8	1,588	1,961E-02
IPI00122362	Protein disulfide isomerase-related protein	Pdia5	Q921X9;Q9CSM8	1,574	2,084E-02
IPI00135660	Cavin-2	Sdpr	Q63918	1,555	2,273E-02
IPI00226430;I	Acetyl-CoA acyltransferase	Acaa2	Q8BWT1;Q3TIT9;Q3UKH3	1,548	4,000E-02
IPI00928513;I	Interferon-related developmental regulator 1	Ifrd1	Q80XM4;P19182;E9Q949;	1,538	2,449E-02
IPI00126072	Vesicle amine transport protein 1 homolog (T ca	Vat1	Q62465;Q3TXD3;Q3U331;	1,527	2,567E-02
IPI00116753	Electron transfer flavoprotein subunit alpha, mit	Etfa	Q99LC5;B1B1B4	1,517	2,687E-02
IPI00128671;I	Cyclin-A2	Ccna2	P51943;Q8BRG1;D6RIK7	1,516	2,694E-02
IPI00129517;I	Peroxiredoxin-5, mitochondrial	Prdx5	P99029-1;P99029;Q3U7H	1,498	2,906E-02
IPI00985904;I	Mediator complex subunit 15	Med15	Q3TE00;E9Q7C1;Q6KAM1	1,464	3,358E-02
IPI00396833;I	Tyrosine--tRNA ligase	Yars2	Q8BYL4	1,432	3,847E-02
IPI00319830;I	Beta-II spectrin	Spnb2	Q62261-1;Q62261;Q8BQ3	1,431	3,858E-02
IPI00170126;I	Pitrilysin metalloproteinase 1	Pitrm1	Q8K411-1;Q8K411;Q8K41	1,407	4,248E-02
IPI00410836;I	Flap endonuclease 1	Fen1	E9PYV9;Q3TGH6;Q8C5X6;	1,397	4,427E-02
IPI00310862;I	Discs large homolog 7	Dlg7	Q8K4R9-1;Q8K4R9;Q8K4R	1,378	4,775E-02
IPI00230395;I	Annexin A1	Anxa1	P10107;B7STB7;Q3U5N9;(	1,373	4,877E-02

Supplementary Table 2

Accession	Protein name	MB1	SNX vs Gapdh10	MB2	SNX vs Gapdh11
Q9JLQ0	CD2-associated protein OS=Mus musculus OX=10090 GN=Cd2ap PE=1 SV=3		3024,58		2120,95
Q9DBC7	cAMP-dependent protein kinase type I-alpha regulatory subunit OS=Mus musculus OX=10090 GN=		1906,24		157,08
Q9CWK8	Sorting nexin-2 OS=Mus musculus OX=10090 GN=Snx2 PE=1 SV=2		1496,56		530,67
P97793	ALK tyrosine kinase receptor OS=Mus musculus OX=10090 GN=Alk PE=1 SV=2		1493,39		1012,87
Q91YL3	Uridine-cytidine kinase-like 1 OS=Mus musculus OX=10090 GN=Uckl1 PE=1 SV=1		1350,58		79,52
B1AVZ0	Uracil phosphoribosyltransferase homolog OS=Mus musculus OX=10090 GN=Uprt PE=1 SV=1		1097,46		225,43
Q9QZQ1	Afadin OS=Mus musculus OX=10090 GN=Afdn PE=1 SV=3		1097,05		1936,26
Q9D8U8	Sorting nexin-5 OS=Mus musculus OX=10090 GN=Snx5 PE=1 SV=1		830,60		2612,74
Q8R050	Eukaryotic peptide chain release factor GTP-binding subunit ERF3A OS=Mus musculus OX=10090 G		790,76		121,09
Q99MR6	Serrate RNA effector molecule homolog OS=Mus musculus OX=10090 GN=Srrt PE=1 SV=1		763,35		33,23
P97496	SWI/SNF complex subunit SMARCC1 OS=Mus musculus OX=10090 GN=Smarcc1 PE=1 SV=2		751,59		31,82
Q62188	Dihydropyrimidinase-related protein 3 OS=Mus musculus OX=10090 GN=Dpysl3 PE=1 SV=1		696,63		631,25
Q9JJG0	Transforming acidic coiled-coil-containing protein 2 OS=Mus musculus OX=10090 GN=Tacc2 PE=1 S		674,03		448,98
P55194	SH3 domain-binding protein 1 OS=Mus musculus OX=10090 GN=Sh3bp1 PE=1 SV=3		570,38		813,22
Q5F226	Protocadherin Fat 2 OS=Mus musculus OX=10090 GN=Fat2 PE=1 SV=1		535,57		1801,48
Q02105	Complement C1q subcomponent subunit C OS=Mus musculus OX=10090 GN=C1qc PE=1 SV=2		484,07		683,38
P32261	Antithrombin-III OS=Mus musculus OX=10090 GN=Serpinc1 PE=1 SV=1		452,50		512,72
P52430	Serum paraoxonase/arylesterase 1 OS=Mus musculus OX=10090 GN=Pon1 PE=1 SV=2		396,00		726,65
Q6PAR5	GTPase-activating protein and VPS9 domain-containing protein 1 OS=Mus musculus OX=10090 GN=		320,22		93,65
Q62086	Serum paraoxonase/arylesterase 2 OS=Mus musculus OX=10090 GN=Pon2 PE=1 SV=2		316,25		442,78
Q9WV80	Sorting nexin-1 OS=Mus musculus OX=10090 GN=Snx1 PE=1 SV=1		289,30		653,97
O08638	Myosin-11 OS=Mus musculus OX=10090 GN=Myh11 PE=1 SV=1		282,33		411,55
Q6ZQ03	Formin-binding protein 4 OS=Mus musculus OX=10090 GN=Fnbp4 PE=1 SV=2		268,95		164,27
P19221	Prothrombin OS=Mus musculus OX=10090 GN=F2 PE=1 SV=1		225,38		389,62
Q7TME2	Sperm-associated antigen 5 OS=Mus musculus OX=10090 GN=Spag5 PE=1 SV=1		210,76		241,07
O88783	Coagulation factor V OS=Mus musculus OX=10090 GN=F5 PE=1 SV=1		196,96		158,54
Q80WV3	Carbohydrate sulfotransferase 2 OS=Mus musculus OX=10090 GN=Chst2 PE=2 SV=3		190,42		265,90
Q99PM9	Uridine-cytidine kinase 2 OS=Mus musculus OX=10090 GN=Uck2 PE=1 SV=1		186,24		5,82
Q8C804	Spindle and centriole-associated protein 1 OS=Mus musculus OX=10090 GN=Spice1 PE=1 SV=2		172,34		171,96
Q9DCZ4	MICOS complex subunit Mic26 OS=Mus musculus OX=10090 GN=Apoo PE=1 SV=2		170,49		227,05
Q9QWY8	Arf-GAP with SH3 domain, ANK repeat and PH domain-containing protein 1 OS=Mus musculus OX=		150,44		166,52
A2RSJ4	UHRF1-binding protein 1-like OS=Mus musculus OX=10090 GN=Uhrf1bp1l PE=1 SV=2		145,76		333,03
P52623	Uridine-cytidine kinase 1 OS=Mus musculus OX=10090 GN=Uck1 PE=1 SV=2		128,67		20,68
Q3UYV9	Nuclear cap-binding protein subunit 1 OS=Mus musculus OX=10090 GN=Ncbp1 PE=1 SV=2		125,81		21,82
P25976	Nucleolar transcription factor 1 OS=Mus musculus OX=10090 GN=Ubtf PE=1 SV=1		115,65		13,47
P27641	X-ray repair cross-complementing protein 5 OS=Mus musculus OX=10090 GN=Xrcc5 PE=1 SV=4		103,47		7,31
Q4VBE8	WD repeat-containing protein 18 OS=Mus musculus OX=10090 GN=Wdr18 PE=1 SV=1		102,56		22,39
P67778	Prohibitin OS=Mus musculus OX=10090 GN=Phb PE=1 SV=1		100,86		47,88
Q8R550	SH3 domain-containing kinase-binding protein 1 OS=Mus musculus OX=10090 GN=Sh3kbp1 PE=1 S		97,79		660,85
Q9DAW6	U4/U6 small nuclear ribonucleoprotein Prp4 OS=Mus musculus OX=10090 GN=Prpf4 PE=1 SV=1		93,53		10,02
Q3UQI9	Probable ubiquitin carboxyl-terminal hydrolase MINDY-4 OS=Mus musculus OX=10090 GN=Mindy4		92,18		276,32
Q9R0I7	YLP motif-containing protein 1 OS=Mus musculus OX=10090 GN=Ylpm1 PE=2 SV=2		89,57		653,10
P06684	Complement C5 OS=Mus musculus OX=10090 GN=C5 PE=1 SV=2		78,73		14,71
P54276	DNA mismatch repair protein Msh6 OS=Mus musculus OX=10090 GN=Msh6 PE=1 SV=3		76,30		10,80
E9Q557	Desmoplakin OS=Mus musculus OX=10090 GN=Dsp PE=1 SV=1		73,25		231,32
Q8K0V4	CCR4-NOT transcription complex subunit 3 OS=Mus musculus OX=10090 GN=Cnot3 PE=1 SV=1		65,70		39,79
Q62191	E3 ubiquitin-protein ligase TRIM21 OS=Mus musculus OX=10090 GN=Trim21 PE=1 SV=1		57,80		19,48
Q99KW3	TRIO and F-actin-binding protein OS=Mus musculus OX=10090 GN=Triobp PE=1 SV=3		56,28		334,02
Q4KWH5	1-phosphatidylinositol 4,5-bisphosphate phosphodiesterase eta-1 OS=Mus musculus OX=10090 GN		54,75		10,68
O08553	Dihydropyrimidinase-related protein 2 OS=Mus musculus OX=10090 GN=Dpysl2 PE=1 SV=2		54,12		59,53
Q811S7	Upstream-binding protein 1 OS=Mus musculus OX=10090 GN=Ubp1 PE=1 SV=1		53,53		7,14
Q91X43	SH3 domain-containing protein 19 OS=Mus musculus OX=10090 GN=Sh3d19 PE=1 SV=2		52,12		63,87
Q4U2R1	E3 ubiquitin-protein ligase HERC2 OS=Mus musculus OX=10090 GN=Herc2 PE=1 SV=3		49,53		282,17
Q3TMW1	Coiled-coil domain-containing protein 102A OS=Mus musculus OX=10090 GN=Ccdc102a PE=1 SV=2		48,72		629,41
P70255	Nuclear factor 1 C-type OS=Mus musculus OX=10090 GN=Nfic PE=1 SV=1		48,06		42,71
P43275	Histone H1.1 OS=Mus musculus OX=10090 GN=Hist1h1a PE=1 SV=2		47,88		66,09
P11103	Poly [ADP-ribose] polymerase 1 OS=Mus musculus OX=10090 GN=Parp1 PE=1 SV=3		45,98		275,15
Q9CQ49	Nuclear cap-binding protein subunit 2 OS=Mus musculus OX=10090 GN=Ncbp2 PE=1 SV=1		45,11		9,92
P97386	DNA ligase 3 OS=Mus musculus OX=10090 GN=Lig3 PE=1 SV=2		43,27		12,20
Q9Z1N5	Spliceosome RNA helicase Ddx39b OS=Mus musculus OX=10090 GN=Ddx39b PE=1 SV=1		41,52		138,49
Q9DBR7	Protein phosphatase 1 regulatory subunit 12A OS=Mus musculus OX=10090 GN=Ppp1r12a PE=1 SV		40,32		223,47
P43247	DNA mismatch repair protein Msh2 OS=Mus musculus OX=10090 GN=Msh2 PE=1 SV=1		39,19		5,66
Q8C4Y3	Negative elongation factor B OS=Mus musculus OX=10090 GN=Nelfb PE=1 SV=2		38,35		56,50
Q3U2K0	Protein FAM193B OS=Mus musculus OX=10090 GN=Fam193b PE=1 SV=2		37,69		114,22
Q9JLC8	Sacsin OS=Mus musculus OX=10090 GN=Sacs PE=1 SV=2		37,66		16,76
Q9DBD5	Proline-, glutamic acid- and leucine-rich protein 1 OS=Mus musculus OX=10090 GN=Pelp1 PE=1 SV=		37,61		265,18
Q640N1	Adipocyte enhancer-binding protein 1 OS=Mus musculus OX=10090 GN=Aebp1 PE=1 SV=1		36,99		31,31
P01942	Hemoglobin subunit alpha OS=Mus musculus OX=10090 GN=Hba PE=1 SV=2		34,96		11,52
Q62159	Rho-related GTP-binding protein RhoC OS=Mus musculus OX=10090 GN=Rhoc PE=1 SV=2		34,69		185,17
P47757	F-actin-capping protein subunit beta OS=Mus musculus OX=10090 GN=Capzb PE=1 SV=3		32,48		16,89
Q91V81	RNA-binding protein 42 OS=Mus musculus OX=10090 GN=Rbm42 PE=1 SV=1		32,34		15,45
Q91VZ6	Stromal membrane-associated protein 1 OS=Mus musculus OX=10090 GN=Smap1 PE=1 SV=1		31,60		18,60
P08207	Protein S100-A10 OS=Mus musculus OX=10090 GN=S100a10 PE=1 SV=2		30,54		8,42
P39447	Tight junction protein ZO-1 OS=Mus musculus OX=10090 GN=Tjp1 PE=1 SV=2		30,47		11,64
P50518	V-type proton ATPase subunit E 1 OS=Mus musculus OX=10090 GN=Atp6v1e1 PE=1 SV=2		30,37		25,75
Q8CGY8	UDP-N-acetylglucosamine--peptide N-acetylglucosaminyltransferase 110 kDa subunit OS=Mus mus		28,22		85,37
P47753	F-actin-capping protein subunit alpha-1 OS=Mus musculus OX=10090 GN=Capza1 PE=1 SV=4		27,33		106,23
Q810A7	ATP-dependent RNA helicase DDX42 OS=Mus musculus OX=10090 GN=Ddx42 PE=1 SV=3		26,06		5,02
P97384	Annexin A11 OS=Mus musculus OX=10090 GN=Anxa11 PE=1 SV=2		25,55		14,79
P33215	Protein NEDD1 OS=Mus musculus OX=10090 GN=Nedd1 PE=1 SV=2		24,87		10,94
Q9CWN7	CCR4-NOT transcription complex subunit 11 OS=Mus musculus OX=10090 GN=Cnot11 PE=1 SV=1		24,83		26,80
P02088	Hemoglobin subunit beta-1 OS=Mus musculus OX=10090 GN=Hbb-b1 PE=1 SV=2		24,58		31,89
Q80XI3	Eukaryotic translation initiation factor 4 gamma 3 OS=Mus musculus OX=10090 GN=Eif4g3 PE=1 SV		23,33		12,29
P02104	Hemoglobin subunit epsilon-Y2 OS=Mus musculus OX=10090 GN=Hbb-y PE=1 SV=2		22,68		25,66
P62141	Serine/threonine-protein phosphatase PP1-beta catalytic subunit OS=Mus musculus OX=10090 GN		21,59		11,16
P70218	Mitogen-activated protein kinase kinase kinase kinase 1 OS=Mus musculus OX=10090 GN=Map4k1		21,55		23,87
Q3UMT1	Protein phosphatase 1 regulatory subunit 12C OS=Mus musculus OX=10090 GN=Ppp1r12c PE=1 SV		21,51		18,11
Q80YR4	E3 ubiquitin-protein ligase ZNF598 OS=Mus musculus OX=10090 GN=Znf598 PE=1 SV=1		21,19		18,22
Q91YR7	Pre-mRNA-processing factor 6 OS=Mus musculus OX=10090 GN=Prpf6 PE=1 SV=1		20,60		7,91
P08113	Endoplasmin OS=Mus musculus OX=10090 GN=Hsp90b1 PE=1 SV=2		20,17		5,06
Q6PCZ4	Melanoma-associated antigen E1 OS=Mus musculus OX=10090 GN=Magee1 PE=1 SV=1		19,06		112,44
P19426	Negative elongation factor E OS=Mus musculus OX=10090 GN=Nelfe PE=1 SV=2		18,86		308,39
Q91VR8	Protein BRICK1 OS=Mus musculus OX=10090 GN=Brk1 PE=1 SV=1		18,86		28,99
P98064	Mannan-binding lectin serine protease 1 OS=Mus musculus OX=10090 GN=Masp1 PE=1 SV=2		18,38		32,32
P68181	cAMP-dependent protein kinase catalytic subunit beta OS=Mus musculus OX=10090 GN=Prkacb PE		18,25		18,04
Q8BZN4	NUAK family SNF1-like kinase 2 OS=Mus musculus OX=10090 GN=Nuak2 PE=1 SV=2		18,00		80,23
Q9D554	Splicing factor 3A subunit 3 OS=Mus musculus OX=10090 GN=Sf3a3 PE=1 SV=2		16,83		29,10
P47754	F-actin-capping protein subunit alpha-2 OS=Mus musculus OX=10090 GN=Capza2 PE=1 SV=3		16,22		82,93
Q02384	Son of sevenless homolog 2 OS=Mus musculus OX=10090 GN=Sos2 PE=1 SV=2		15,17		12,15
E9QAM5	Helicase with zinc finger domain 2 OS=Mus musculus OX=10090 GN=Helz2 PE=1 SV=1		14,54		7,01
A2AF47	Dedicator of cytokinesis protein 11 OS=Mus musculus OX=10090 GN=Dock11 PE=1 SV=1		14,04		83,58
P58021	Transmembrane 9 superfamily member 2 OS=Mus musculus OX=10090 GN=Tm9sf2 PE=1 SV=1		13,72		22,64
Q3TZX8	Polynucleotide 5'-hydroxyl-kinase NOL9 OS=Mus musculus OX=10090 GN=Nol9 PE=1 SV=1		13,70		33,27
O70274	Protein tyrosine phosphatase type IVA 2 OS=Mus musculus OX=10090 GN=Ptp4a2 PE=1 SV=1		13,68		8,96
Q61136	Serine/threonine-protein kinase PRP4 homolog OS=Mus musculus OX=10090 GN=Prpf4b PE=1 SV=		13,41		13,16
Q9CY57	Chromatin target of PRMT1 protein OS=Mus musculus OX=10090 GN=Chtop PE=1 SV=2		13,14		31,22
Q9Z2K1	Keratin, type I cytoskeletal 16 OS=Mus musculus OX=10090 GN=Krt16 PE=1 SV=3		12,90		6,01
Q9JJ80	Ribosome production factor 2 homolog OS=Mus musculus OX=10090 GN=Rpf2 PE=2 SV=2		12,68		8,70
Q61990	Poly(rC)-binding protein 2 OS=Mus musculus OX=10090 GN=Pcbp2 PE=1 SV=1		12,52		9,16
Q91WN1	DnaJ homolog subfamily C member 9 OS=Mus musculus OX=10090 GN=Dnajc9 PE=1 SV=2		12,35		6,53
Q921Q7	Ras and Rab interactor 1 OS=Mus musculus OX=10090 GN=Rin1 PE=1 SV=1		12,29		12,55
Q9Z315	U4/U6.U5 tri-snRNP-associated protein 1 OS=Mus musculus OX=10090 GN=Sart1 PE=1 SV=1		11,30		7,03
Q62203	Splicing factor 3A subunit 2 OS=Mus musculus OX=10090 GN=Sf3a2 PE=1 SV=2		11,10		10,83
Q8BG30	Negative elongation factor A OS=Mus musculus OX=10090 GN=Nelfa PE=1 SV=1		11,02		10,01
Q60930	Voltage-dependent anion-selective channel protein 2 OS=Mus musculus OX=10090 GN=Vdac2 PE=		10,90		5,91
Q8K1N4	Spermatogenesis-associated serine-rich protein 2 OS=Mus musculus OX=10090 GN=Spats2 PE=1 SV		10,87		9,51
P97434	Myosin phosphatase Rho-interacting protein OS=Mus musculus OX=10090 GN=Mprip PE=1 SV=2		10,45		16,65
Q9CY27	Very-long-chain enoyl-CoA reductase OS=Mus musculus OX=10090 GN=Tecr PE=1 SV=1		9,99		8,68
E9Q1P8	Interferon regulatory factor 2-binding protein 2 OS=Mus musculus OX=10090 GN=Irf2bp2 PE=1 SV=		9,99		31,05
Q6Y685	Transforming acidic coiled-coil-containing protein 1 OS=Mus musculus OX=10090 GN=Tacc1 PE=1 S		9,97		24,61
Q921M3	Splicing factor 3B subunit 3 OS=Mus musculus OX=10090 GN=Sf3b3 PE=1 SV=1		9,84		5,69
A2AMM0	Caveolae-associated protein 4 OS=Mus musculus OX=10090 GN=Cavin4 PE=1 SV=1		9,82		16,96
Q02819	Nucleobindin-1 OS=Mus musculus OX=10090 GN=Nucb1 PE=1 SV=2		9,65		74,93
Q0P678	Zinc finger CCCH domain-containing protein 18 OS=Mus musculus OX=10090 GN=Zc3h18 PE=1 SV=		9,43		9,72
Q9EP71	Ankycorbin OS=Mus musculus OX=10090 GN=Rai14 PE=1 SV=1		9,08		12,43
P29391	Ferritin light chain 1 OS=Mus musculus OX=10090 GN=Ftl1 PE=1 SV=2		8,99		11,83
Q62556	Butyrophilin subfamily 1 member A1 OS=Mus musculus OX=10090 GN=Btn1a1 PE=1 SV=2		8,91		100,55
Q6ZQ08	CCR4-NOT transcription complex subunit 1 OS=Mus musculus OX=10090 GN=Cnot1 PE=1 SV=2		8,91		10,81
Q80TY0	Formin-binding protein 1 OS=Mus musculus OX=10090 GN=Fnbp1 PE=1 SV=2		8,76		9,32
O54988	STE20-like serine/threonine-protein kinase OS=Mus musculus OX=10090 GN=Slk PE=1 SV=2		7,98		18,48
Q9CW03	Structural maintenance of chromosomes protein 3 OS=Mus musculus OX=10090 GN=Smc3 PE=1 SV		7,98		8,27
Q923Z0	G-protein coupled receptor family C group 5 member B OS=Mus musculus OX=10090 GN=Gprc5b P		7,92		359,79
Q9WV55	Vesicle-associated membrane protein-associated protein A OS=Mus musculus OX=10090 GN=Vapa		7,92		7,23
Q149F3	Eukaryotic peptide chain release factor GTP-binding subunit ERF3B OS=Mus musculus OX=10090 G		7,91		24,52
Q922L6	Negative elongation factor D OS=Mus musculus OX=10090 GN=Nelfcd PE=1 SV=2		7,88		42,41
Q8BFX3	BTB/POZ domain-containing protein KCTD3 OS=Mus musculus OX=10090 GN=Kctd3 PE=1 SV=1		7,61		11,43
P07356	Annexin A2 OS=Mus musculus OX=10090 GN=Anxa2 PE=1 SV=2		7,49		6,42
Q9ES28	Rho guanine nucleotide exchange factor 7 OS=Mus musculus OX=10090 GN=Arhgef7 PE=1 SV=2		7,43		22,32
P29788	Vitronectin OS=Mus musculus OX=10090 GN=Vtn PE=1 SV=2		7,35		22,23
Q9JKY0	CCR4-NOT transcription complex subunit 9 OS=Mus musculus OX=10090 GN=Cnot9 PE=1 SV=1		6,82		64,85
Q8C5L3	CCR4-NOT transcription complex subunit 2 OS=Mus musculus OX=10090 GN=Cnot2 PE=1 SV=2		6,77		19,82
Q9CQQ4	Gem-associated protein 2 OS=Mus musculus OX=10090 GN=Gemin2 PE=2 SV=1		6,40		18,74
P28660	Nck-associated protein 1 OS=Mus musculus OX=10090 GN=Nckap1 PE=1 SV=2		6,06		6,65
Q60596	DNA repair protein XRCC1 OS=Mus musculus OX=10090 GN=Xrcc1 PE=1 SV=2		5,84		8,38
Q3TKT4	Transcription activator BRG1 OS=Mus musculus OX=10090 GN=Smarca4 PE=1 SV=1		5,69		6,69
Q8R0X7	Sphingosine-1-phosphate lyase 1 OS=Mus musculus OX=10090 GN=Sgpl1 PE=1 SV=1		5,54		9,17
Q8VED5	Keratin, type II cytoskeletal 79 OS=Mus musculus OX=10090 GN=Krt79 PE=1 SV=2		5,52		5,93
P84084	ADP-ribosylation factor 5 OS=Mus musculus OX=10090 GN=Arf5 PE=1 SV=2		5,38		8,45
Q80SW1	S-adenosylhomocysteine hydrolase-like protein 1 OS=Mus musculus OX=10090 GN=Ahcyl1 PE=1 SV		5,27		6,73

Supplementary Table 3

Table S4. Primers for site-direct mutagenesis.

Primer Name	Oligonucleotide sequence (5’-3’)
MuRF2 C42S His/Myc for	GCCTGTGGTCATTCTCCCTAGCCAGCACAA
MuRF2 C42S His/Myc rev	TTCGTGAACATCTCTAGGCAGATGGGACAG
MuRF2 C50S His/Myc for	GCACAACCTGTGCAGGAAAAGTGCCAGTGACATC
MuRF2 C50S His/Myc rev	TGGCAAGGGAGAATGACCACAGGCTTCGTGAACA
MuRF3 C42S His/Myc for	CCCGTGGTGATCTTGCCCAGCCAACACAAC
MuRF3 C42S His/Myc rev	CTTGGAGAACATCTCCAGGCAGATGG
MuRF3 C50S His/Myc for	CTGTGCCGCAAGAGTGCCAACGACGTCTTC
MuRF3 C50S His/Myc rev	GTTGTGTTGGCAGGGCAAGATCACCACGGG
SNX5 K290R for	GTCTCATCAGATGAAGACTTAAGACTGACAGAGCTCCTCCGATAC
SNX5 K290R rev	GTATCGGAGGAGCTCTGTCAGTCTTAAGTCTTCATCTGATGAGAC
SNX5 K324R for	GACTATGAGAATTCAAACAGAGCTTTGGACAAGGCCCGG
SNX5 K324R rev	CCGGGCCTTGTCCAAAGCTCTGTTTGAATTCTCATAGTC

Table S5. Primers for generation of retroviral expression plasmid.

Primer Name	Oligonucleotide sequence (5’-3’)
SNX5 His/Myc for (NotI)	ATAAGAATGCGGCCGCATTCTTATGGCCGCGGTTC
SNX5 His/Myc rev (NotI)	ATAGTTTAGCGGCCGCTCAATGATGATGATGATGATG

Table S6. Primers for quantitative real-time PCR.

Primer Name	Oligonucleotide sequence (5’-3’)
Mm_Snx5 for	GTTCCCGAGTTGCTGGAG
Mm_Snx5 rev	GCGATGGGTCAACATTCAG
Mm_Prkar1a for	TGATGCTATGTTTCCAGTCTCC
Mm_Prkar1a rev	CAATCACATAGAAGTTATCCCCTTC
Mm_Mymk for	ATCGCTACCAAGAGGCGTT
Mm_Mymk rev	CACAGCACAGACAAACCAGG
Mm_Mymx for	CAGGAGGGCAAGAAGTTCAG
Mm_Mymx rev	ATGTCTTGGGAGCTCAGTCG
Mm_Myog for	GACTTGACCTTGGACCTTGG
Mm_Myog rev	CGCTGTGGGAGTTGCATT
Mm_Ache for	GGGCTCCTACTTTCTGGTTTAC
Mm_Ache rev	TTCAGGTTCAGGCTCACATATT
Mm_Hdac5 for	GCATGAACTCTCCCAACGAG
Mm_Hdac5 rev	TTCACCTCCACTGCCACAG
Mm_Myh1 for	GAAGATGTTCCTGTGGATGG
Mm_Myh1 rev	TCGTTGGTGAAGTTGATGC
Mm_Myh2 for	AACTCCAGGCAAAAGTGAAATC
Mm_Myh2 rev	TGGATAGATTTGTGTTGGATTGTT
Mm_Myh3 for	AGTAGCCAGGATGGGAAAGTC
Mm_Myh3 rev	GTCCTCTGGCTTAACCACCA
Mm_Myh4 for	GGGAACATGAAATTCAAGCAA
Mm_Myh4 rev	ATAGGCAGCCTTGTCAGCAA
Mm_Myh7 for	CGCATCAAGGAGCTCACC

Mm_Myh7 rev	CTGCAGCCGCAGTAGGTT
Mm_Mstn for	AGGGCAGTGAGAGAGAAGAA
Mm_Mstn rev	GTTTCCGTGGTAGCGTGATAA
Mm_Mstn-promoter for	ACAGCACTCCAAGTCTTAAAGG
Mm_Mstn- promoter rev	TCACAAGTCACCAAGCAGTATT
Mm_RPL30 for	TGGTGTTTGACGCTCTGG
Mm_RPL30 rev	GTTGGAGCCTAGAGTTGATCG
Mm_Gapdh for	GATCAAACGCTTGCGAATCT
Mm_Gapdh rev	ATGGTGAAGGTCGGTGTGA

Table S7. nano-LC MS/MS and search parameters

A. Description of the LC-MS/MS experiment settings – Ultimate-QExactive Plus configuration

**nanoLC-parameters for the chromatographic separation of the peptides**	
Equipment	Ultimate 3000 (Thermo Electron, Bremen, Germany)
Trap column	Acclaim PepMap 100-C18 trap column (2cm x75µm, 3µm, 100Å) Thermo Fisher Scientific Inc., Idstein, Germany
Analytical column	Accucore 150-C18 column (25cm x 2.6µm, 2.6µm, 150Å) Thermo Fisher Scientific Inc., Idstein, Germany
Buffer system	0.1% acetic acid, 5% ACN in water (buffer A) and 100% ACN in 0.1% acetic acid (buffer B)
Flow rate	300nL/min
Gradient	linear gradient of buffer B from 5% up to 25% for 120 min
Column oven temperature	40°C
**Mass Spectrometry**	
Equipment	QExactive Plus
Ion source	FlexMap Ion source (Thermo Scientific)
Fragmentation	high-energy collision dissociation (HCD)
Charge state screening	positive

**DDA acquisition**	
**Full MS**	
MS scan resolution	70,000
AGC target	3 x 10E6
Maximum ion injection time for the MS scan	120 ms
MS full scan range	300 to 1650 m/z
Spectra data type	profile
**dd-MS2**	
Resolution	17,500
AGC target	2 x 10E5
Maximum ion injection time for the MS2 scan	120 ms
Selection	Top10 z= +2 to +6 charge state
Isolation width	3.0 m/z
Scan range	200 to 2,000 m/z
Fixed first mass	100 m/z
Spectrum data type	centroid
Minimum AGC target	1 x 10E4
Intensity threshold	8.3 x 10E4
Monoisotopic precursor selection rejected	+1 and +7, +8, and >+8 charged ions
Dynamic exclusion	30 s
Normalized collision energy	27.5eV

B. Presentation of Protein Identification Results

**Protein identification/quantitation**

**Search parameters**	**Settings**
Name of peaklist-generating software and release version (number or date)	Proteome discoverer 2.3 (Thermo Scientific) using SequestHT as search engine
Protein database	Uniprot/Swissprot database limited to murine entries (version 11_2019)
Enzyme specificity considered	Fully tryptic
Precursor mass tolerance	10 ppm
Fragment mass tolerance	0.02 Da
# of missed cleavages permitted	2
Static modification	carbamidomethylation at cysteine
Variable modification	oxidation at methionine; acetylation at protein N-terminus
FDR (peptide level)	1%
Data quantification	MS1 peak area of precursor

Supplementary Figures

Figure S1. A SILAC-AP-MS approach identified the novel MuRF3 interaction partner SNX5. (A) Workflow of the SILAC-AP-MS approach. (B) Western blot analysis of proteins isolated from different murine organs as indicated. (C) qRT-PCR analysis of *Snx5* in different murine organs as indicated. *Snx5* mRNA expression was normalized to *Gapdh*.

Figure S2. MuRF3 reduces MuRF2-dependent SNX5 degradation. (A) C2C12 cells were cotransfected with increasing amounts of MuRF2-[(C42S;C50S)]-Myc(His)6, and a constant amount of SNX5-FLAG. Cells were lysed 24 h later and overexpressed proteins were analyzed by Western blot using anti-Myc and anti-FLAG antibodies. (B) Western blot analysis of C2C12 cells co-transfected with SNX5-FLAG, MuRF2- or increasing amounts of MuRF3-Myc(His)$_6$ as indicated.

Figure S3. MuRF2-mediated reduction in SNX5 stability is mediated by its K290 and K324. (A) PyMOL visualization of putative ubiquitin chain binding sites on SNX5 (PDB 5tp1D) at K290 and K324 (indicated in red). (B) Schematic showing SNX5 ubiquitination sites and K290R, K324R, K290/324R mutants. (C) Cycloheximide (CHX) chase assay in COS7 cells. MuRF2-Myc(His)$_6$ was co-transfected with either SNX5-FLAG or each of K290R, K324R, K290/324R mutants for 48 hours. Cells were then treated with CHX for indicated time points prior to lysis. Proteins were analyzed by Western blot using the indicated antibodies.

Figure S4. SNX5 stabilizes RI-α upon PKA activation. (A) Workflow for identification of SNX5 cargo proteins via mass spectrometry. (B) Sucrose gradient fractionation of PNS isolated from C2C12 cells followed by Western blotting with the indicated antibodies. Individual fractions are indicated. (C) DSS cross-linking of core RI-α oligomers detected via Western blot in vehicle- or Bt2cAMP-treated C2C12 cells. Densitometrical analysis (right panel) was carried out in Image Lab^{TM} software. PKA tetramer-to-RI-α ratios are shown. (D) Immunofluorescence using anti-RI-α (green) and anti-EEA1 (red) antibody in vehicle- or Bt2cAMP and CHX treated NT-WT and SNX5-KO C2C12 myoblasts for 1 or 4 hours. Nuclei were stained with DAPI.

Scale bar, 20 μm. (E) Workflow to enrich endosomal fractions from SNX5-KO and NT-WT myocytes. (F) Work flow of protease protection assay. (G) PNGase F digestion assay, SNX5-coated endosomes isolated from C2C12 cells by IP SNX5 were digested with PNGase F. Western blot with anti-RI-α antibody was performed.

Figure S5. PKA activation is accompanied by an increase in *Prkar1a* expression in SNX5-KO myocytes. qRT-PCR analysis of *Prkar1a* expression in NT-WT and SNX5-KO cells treated with either vehicle or Bt2cAMP for the indicated time points. *Prkar1a* mRNA expression was normalized to *Gapdh*. Statistical significance was determined using one-way ANOVA followed by Tukey's post-hoc test. *$P < 0.05$, **$P < 0.01$, ***$P < 0.001$, ****$P < 0.0001$.

Figure S6. Reintroduction of SNX5 in SNX5-KO myocytes increases the stability of RI-α. Western blot analysis of eGFP or SNX5-Myc(His)$_6$ transduced SNX5-KO myoblasts treated with Bt2cAMP and CHX for indicated time points prior to cell lysis. Densitometric analysis of RI-α levels was carried out in Image Lab™ Software, with the "0-hour" intensity of RI-α set as 1. Protein amounts of RI-α were normalized to GAPDH and RI-α-to-GAPDH ratios per indicated time point are shown.

Figure S7. SNX5 stabilizes RI-α upon PKA activation. (A, B) Western blot of NT-siRNA or SNX5-siRNA transfected C2C12 myoblasts (A) and myotubes (B) co-treated with Bt2cAMP, CHX, and the inhibitors as indicated for 4 hours.

Figure S8. Reduction in free RI-α upon PKA activation is mediated by proteasomal protein degradation in SNX5-HET cells. Western blot analysis of NT-WT and SNX5-HET C2C12 myoblasts treated with Bt2cAMP, CHX, and one of the indicated inhibitors (MG132, CQ, or BafA1) for 4 hours prior to cell lysis.

Figure S9. SNX5 via regulation of PKA activity contributes to myogenic differentiation. (A) qRT-PCR analysis of *Hdac5* mRNA expression from C2C12 cells treated with Bt2cAMP for different time points. *Hdac5* mRNA expression was normalized to *Gapdh*. Data were

analyzed with one-way ANOVA followed by Tukey's post-hoc test. ****$P < 0.0001$. (B) Nuclear and cytoplasmic fractionation followed by Western blot analysis of NT-WT and SNX5-KO C2C12 cells with indicated antibodies. (C) Western blot analysis of vehicle or recombinant myostatin treated C2C12 myotubes (MT3) for 3 days.

A

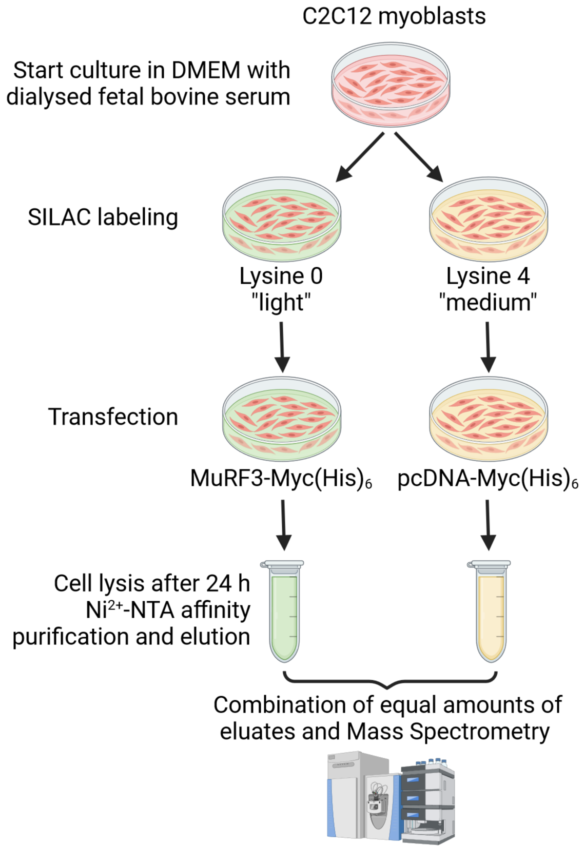

B

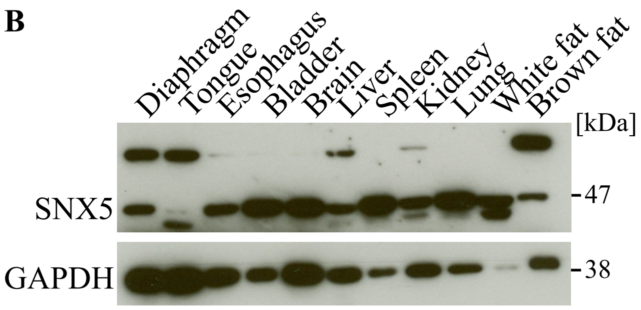

C

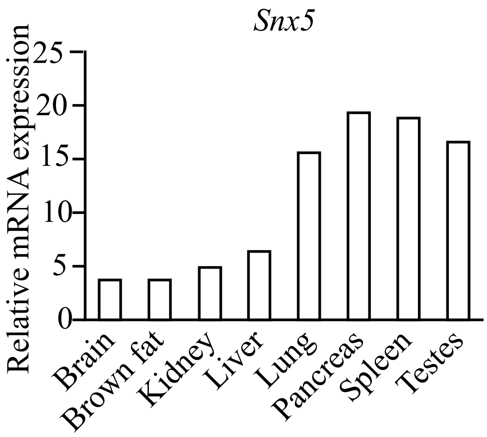

Supplementary Figure 1

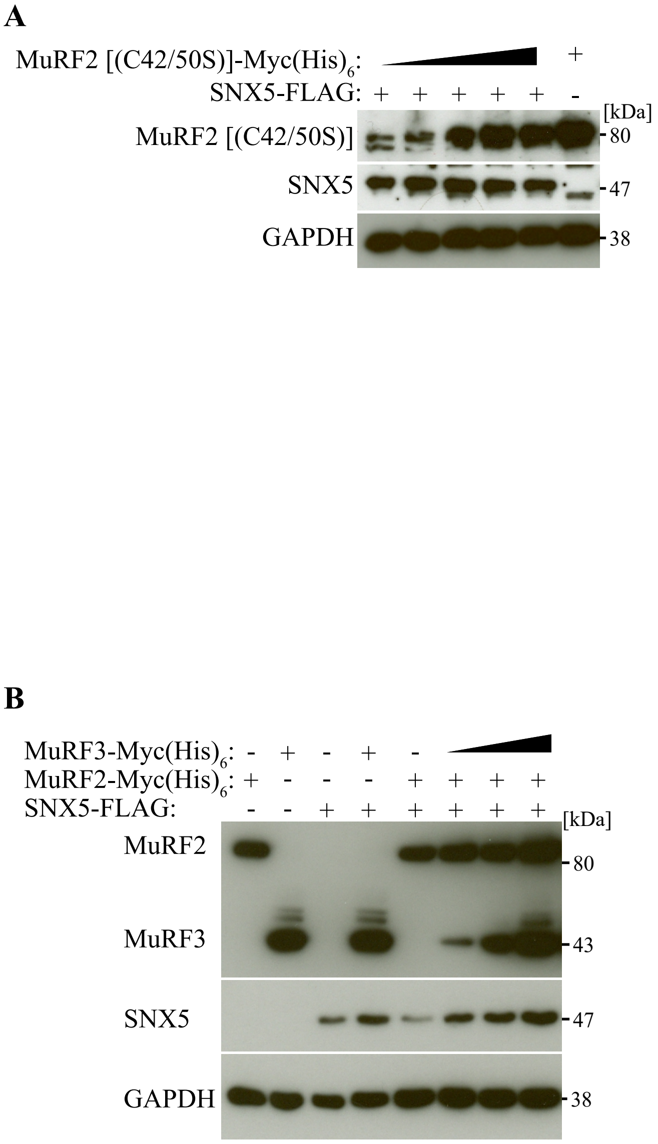

Supplementary Figure 2

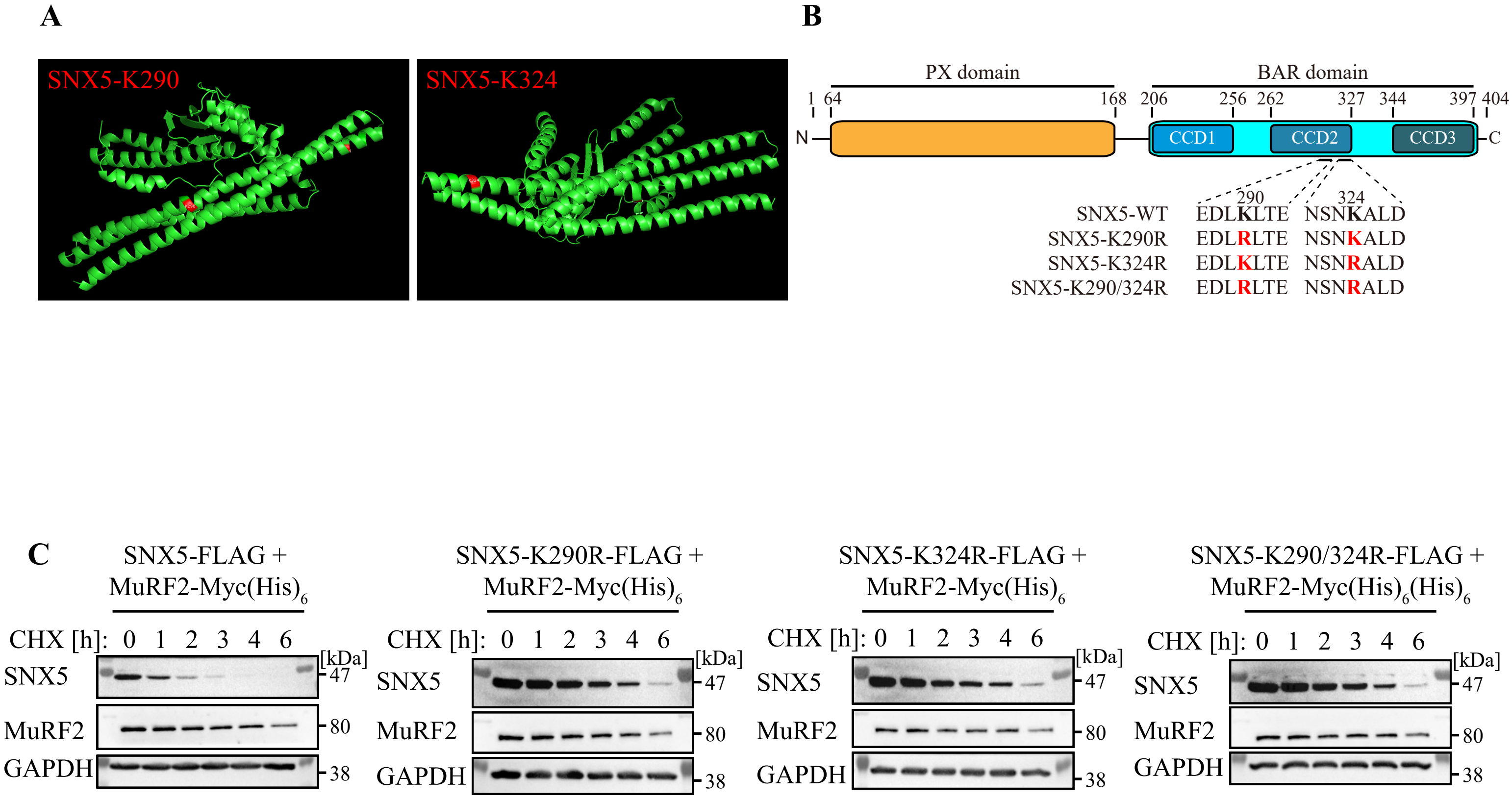

Supplementary Figure 3

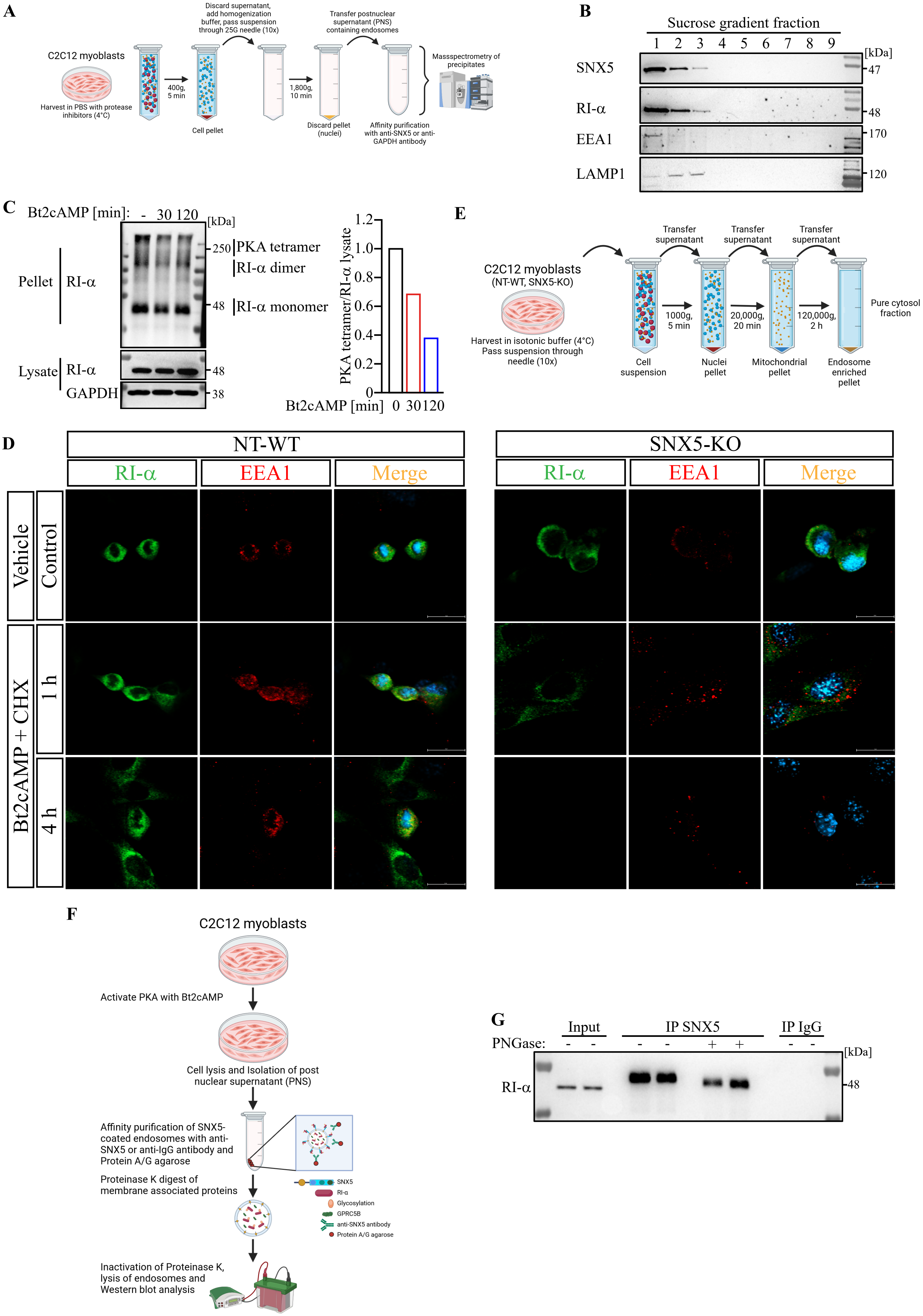

Supplementary Figure 4

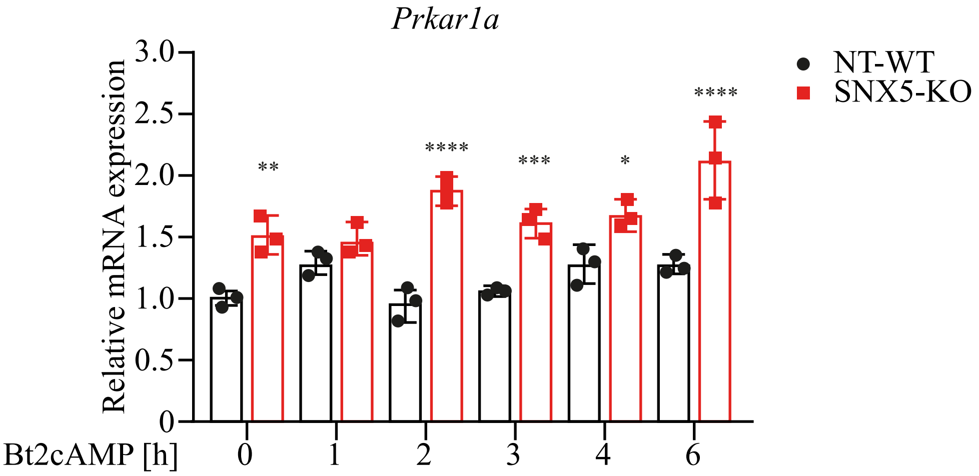

Supplementary Figure 5

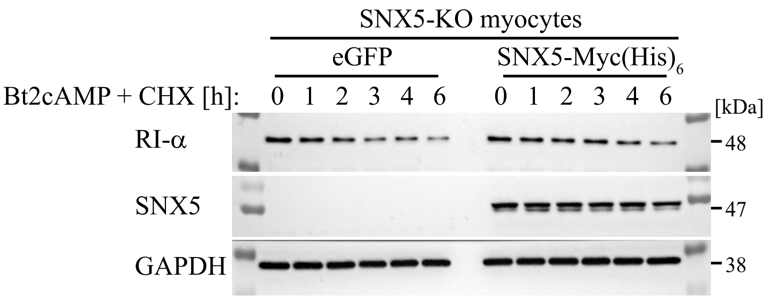

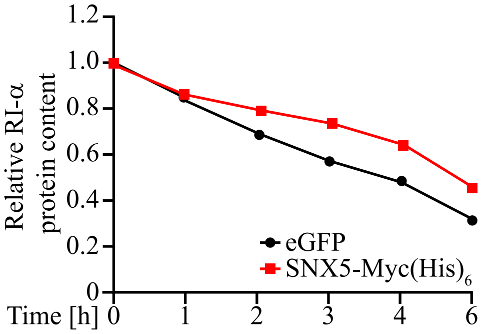

Supplementary Figure 6

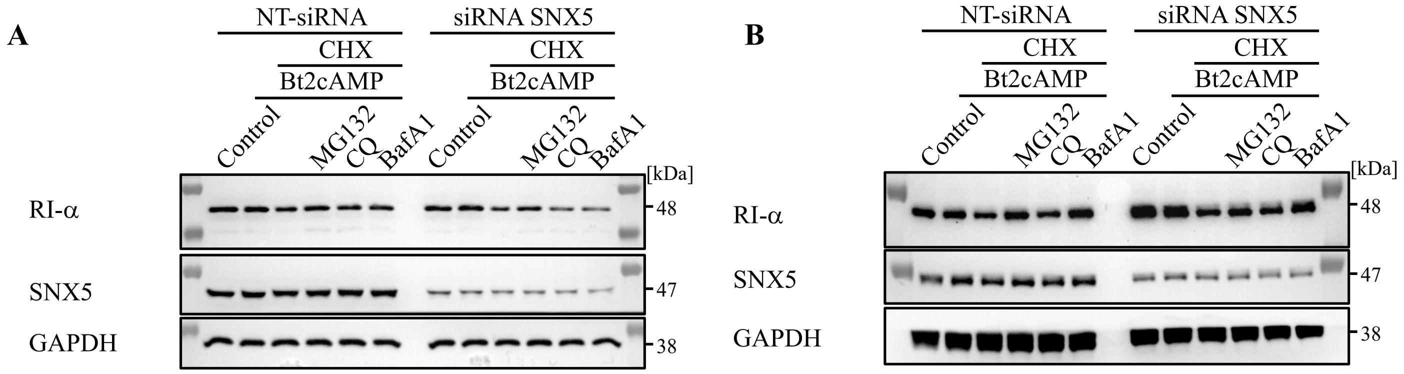

Supplementary Figure 7

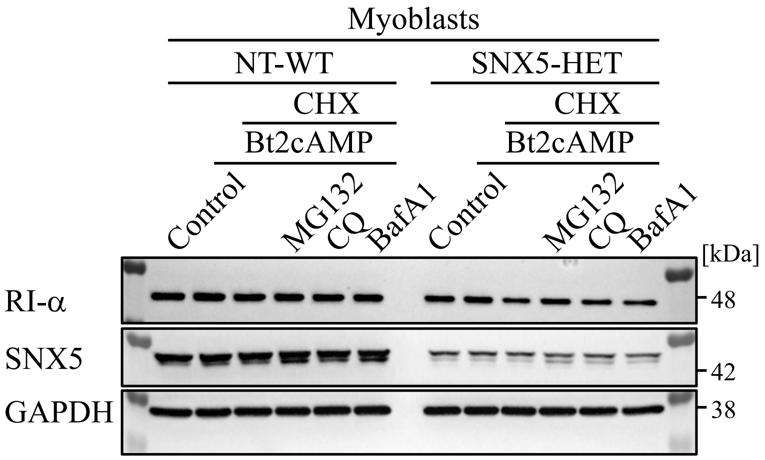

Supplementary Figure 8

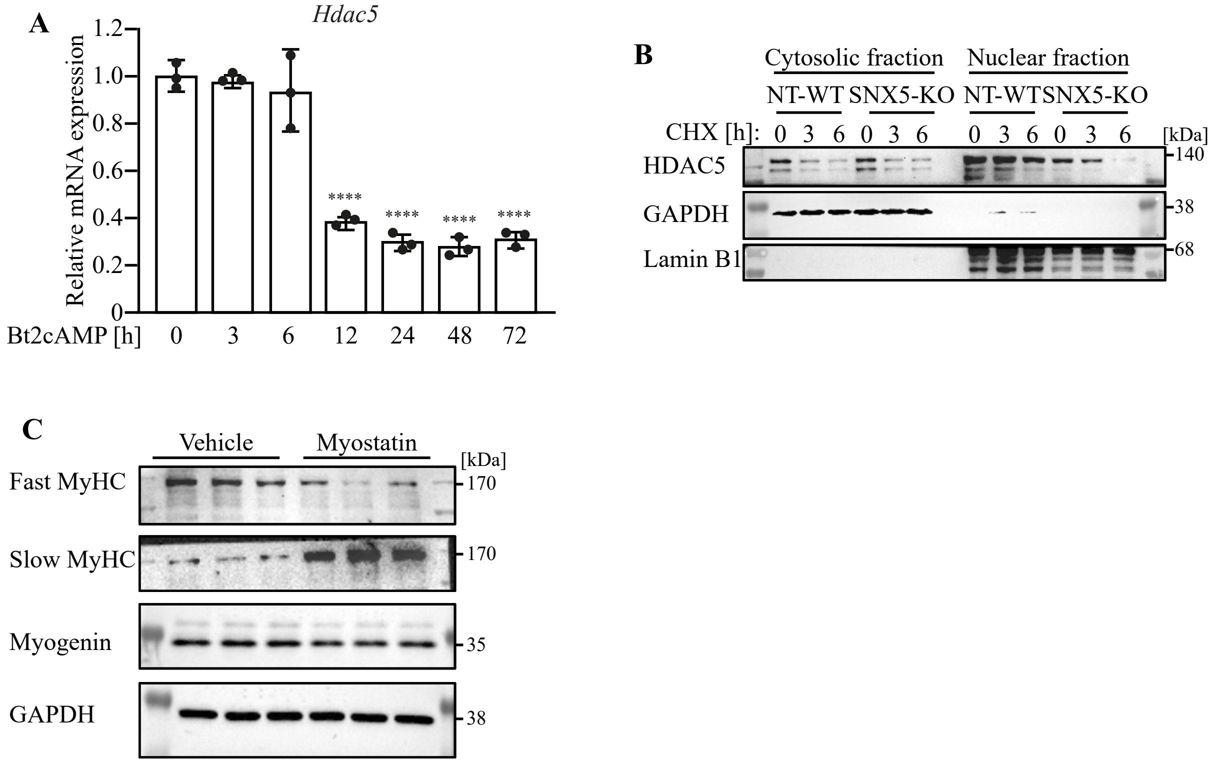

Supplementary Figure 9
